# Taphonomic criteria for identifying Iberian lynx dens in quaternary deposits

**DOI:** 10.1038/s41598-020-63908-6

**Published:** 2020-04-29

**Authors:** Antonio Rodríguez-Hidalgo, Montserrat Sanz, Joan Daura, Antonio Sánchez-Marco

**Affiliations:** 1 Departamento de Prehistoria, Historia Antigua y Arqueología, Facultad de Geografía e Historia, Universidad Complutense de Madrid, Madrid, 28040 Spain; 2 Instituto de Evolución en África (IDEA), Madrid, 28010 Spain; 3 Institut Català de Paleoecologia Humana i Evolució Social (IPHES), Tarragona, 43007 Spain; 4 Grup de Recerca del Quaternari (GRQ)-SERP, Departament d’Història i Arqueologia, Universitat de Barcelona, Barcelona, 08001 Spain; 5 Institut Català de Paleontologia Miquel Crusafont, Campus de la UAB, Bellaterra, 08193 Spain

**Keywords:** Environmental sciences, Palaeontology, Archaeology

## Abstract

For decades, taphonomists have dedicated their efforts to assessing the nature of the massive leporid accumulations recovered at archaeological sites in the northwestern Mediterranean region. Their interest lying in the fact that the European rabbit constituted a critical part of human subsistence during the late Pleistocene and early Holocene. However, rabbits are also a key prey in the food webs of Mediterranean ecosystems and the base of the diet for several specialist predators, including the Iberian lynx (*Lynx pardinus*). For this reason, the origin of rabbit accumulations in northwestern Mediterranean sites has proved a veritable conundrum. Here, we present the zooarchaeological and taphonomic study of more than 3000 faunal and 140 coprolite remains recovered in layer IIIa of Cova del Gegant (Catalonia, Spain). Our analysis indicates that this layer served primarily as a den for the Iberian lynx. The lynxes modified and accumulated rabbit remains and also died at the site creating an accumulation dominated by the two taxa. However, other agents and processes, including human, intervened in the final configuration of the assemblage. Our study contributes to characterizing the Iberian lynx fossil accumulation differentiating between the faunal assemblages accumulated by lynxes and hominins.

## Introduction

The Iberian lynx (*Lynx pardinus*) is a felid species that used to be present throughout the Iberian Peninsula and southern France. Over the last century, the population declined and today it is to be found in only very limited areas of the Iberian Peninsula, primarily in the south^1^. The main prey of the Iberian lynx is the European rabbit (*Oryctolagus cuniculus*), a diet that is supplemented by birds, such as the red-legged partridge (*Alectoris rufa*) and ducks (Anseriformes), small mammals, such as rodents, and sporadically by wild small ungulates, such as red deer fawns or young fallow deer^2^. However, actualistic studies of lynx food habits could be conditioned by the intensive anthropic pressure on the landscape, which means the food preferences of the lynx in the past are not readily evaluated. Insights should usefully be gained from the study of Pleistocene contexts characterised by low human pressure, such as that prevailing at the Cova del Gegant site.

Accumulations of rabbit remains, the main prey of lynx, are very common at archaeological sites. For decades, archaeologists have dedicated their efforts to assessing the nature of these massive leporid accumulations at prehistoric sites in the northwestern Mediterranean (Iberia and Southern France). This field of study is of particular importance for prehistorians given that the rabbit also constituted a critical part of human subsistence during the Upper Palaeolithic and Mesolithic^3–7^. Indeed, some researchers have proposed that the consumption of fast, small game (especially leporids, but also birds) was more common prior to the late Palaeolithic than previously thought and that archaic hominins from the northwestern Mediterranean, as early as Marine Isotope Stage (MIS) 13, had broader diets than those from adjacent regions^8^. However, the origin of rabbit accumulations at Iberian archaeological sites has proved a veritable conundrum.

The fact that the European rabbit (*Oryctolagus cuniculus*) is a key mammal prey species in the food webs of Mediterranean ecosystems^9^ greatly hinders attempts to unravel the taphonomic history of many assemblages. Indeed, most Iberian predators consume rabbit as a major part of their diets, being the main prey for almost 30 raptors and mammalian carnivores in Iberia^10^. Certain Iberian endemic predators, such as the Iberian lynx (*Lynx pardinus*) and the Iberian imperial eagle (*Aquila adalberti*), are even hyper-specialists, with rabbit constituting 100% of their diet for much of the seasonal cycle^2,11^. This means that all these rabbit predators are potential taphonomic agents and bone accumulators of assemblages dominated by their prey. At the same, however, although the European rabbit is highly adaptable, presenting considerable behavioural and ecological plasticity^12,13^, they often live in concentrations of dozens of individuals in large, deep communal warrens in which their populations are more prosperous and their breeding success greater than in shallow or small breeding stops^14^. As result, in large warrens natural dead are originators of large natural accumulations of skeletal remains of these animals^e.g. 15^.

Faced with such a variety of possible modifiers and accumulators of rabbit remains, neo-taphonomic studies have focused on characterizing the taphonomic signals of different rabbit predators^16–33^. Based on anatomical representations, percentage of adults, the fragmentation of bones and modifications on bone surfaces, great progress has been made. And, albeit to a lesser extent, some natural accumulations have also been characterized (based on the recovery of remains from rabbit warrens), in attempts at characterizing intrusive accumulations^15^. However, the results are often frustrating due to the ambiguity of some proxies (e.g. age of leporids)^23^ and, especially, because most Pleistocene assemblages are cumulative palimpsests in which several agents and processes are usually involved, including abundant natural intrusions^8,34–40^.

In this paper, we present a multiproxy study of the faunal assemblage from layer IIIa at the Cova del Gegant site (Catalonia, Spain) dated to MIS 3. We examined all macro-mammal, meso-vertebrate and coprolite remains in terms of their archaeological taphonomy and performed a multivariate statistical analysis. Our results provide new and valuable information for characterizing the leporid accumulations of the Iberian lynx (*Lynx pardinus*), an omnipresent carnivore in the Iberian faunal assemblages of the Pleistocene. Moreover, our study contributes to differentiating faunal assemblages accumulated by lynxes and hominins with a particular focus on rabbits.

## Site stratigraphy

**Figure 1:**
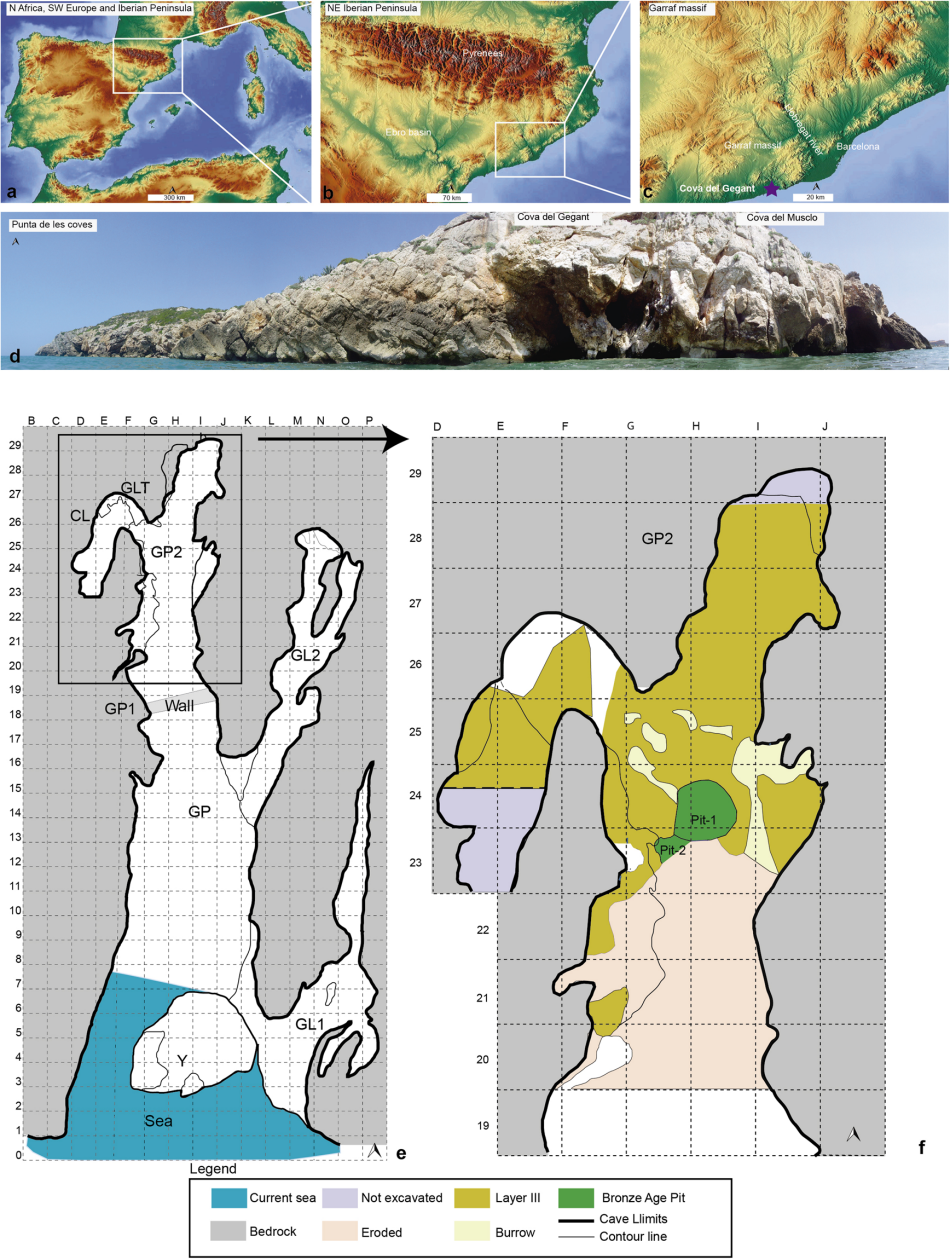
Location and map of Cova del Gegant site. (**a–c**) Location of Cova del Gegant in south-western Europe, NE of Iberian Peninsula and the Garraf massif. Data extracted from Map Tile 4_8–5 (CC BY-SA). OpenStreetMap© licensed under ODdL 1.0 (https://www.openstreetmap.org/copyright) by the OpenStreetMap Foundation (OSMF). ©OpenStreetMap contributors (https://www.openstreetmap.org/). (**d**) Panoramic view of Cova del Gegant and Punta de les coves (Photo by M. Sanz & J. Daura). (**e, f**) Site plan of Cova del Gegant indicating the position of the different galleries discussed in the text and the location of layer III.

Cova del Gegant (Sitges, Barcelona) is a cave located on the seaward edge of the Garraf massif in the central part of the Catalan Coastal Range (1°46′27.33′′E, 41°13′24.75′′ N). The site consists of a principal chamber (GP), partially flooded by the sea and eroded by the ongoing coastal erosion, and an inner area (GP2 and GL-T), whose preserved sedimentation was the target of current fieldwork (Fig. 1).

At least eight site formation episodes, formed by several stratigraphic layers, from the Upper Pleistocene (Episodes 0–3) to the Holocene (Episodes 4–7) have been recognized in the Cova del Gegant stratigraphic sequence. Five Neandertal specimens have been identified^41,42^ and two of these fossils have been recovered in stratigraphic context (layer V), at the base of this sequence (GP2). The archaeological remains discussed in this study come from layer IIIa preserved at the back of the main gallery (GP2). This layer corresponds to Episode 3 and can be chronologically located in MIS 3, a period between 59 and 29 calendar (cal) kyr BP (ca. 56 to 25^14C^ kyr BP). At this point in our study, the chronological framework of this layer is between ca. 31.1 and 30.5 ka cal. BP.

Layer IIIa corresponds to Episode 3a (Fig. 1). It is subjacent to layer II and is located in the inner area of the main gallery (GP2) and formed by light reddish-brown (5YR 6/4) sandy silt containing faunal remains and charcoals^43^. The layer is 10 to 20 cm thick and is located 100–120 cm below datum. Preliminary studies of this layer focused on the archaeological materials from the first excavation (GP2) (years 2007–2010), located between rows 22 and 26^44^.

According to this work, small carnivores were proposed as the principal agents involved in the formation of layer IIIa and that geological and diagenetic processes were primarily responsible for bone modification. No biological modifications were observed, although only a small proportion of the macromammals were examined. In addition, human activity, in the form of hearths, and bioturbation were identified as being responsible for the final configuration of the assemblage. These processes and agents have been chronologically sequenced: thus, the first event can be identified as “the use of the cave as a den/shelter by a small felid or canid, resulting in the accumulation of carnivore bones and coprolites. The second event was both a sporadic, and probably single, occupation of the cave by human groups that left behind the few burned remains and hearths found to date. The third event was the partial reworking of the sediments produced by carnivore activity, as suggested by fragments of coprolites observed in thin section, and by burrowing animals, as evidenced during the excavation. The mixing, and the following sedimentary diagenesis, produced the current patchy distribution presented by the uppermost layers in the combustion structure”^44^ p.112. Our latest study (reported here) examines this interpretation in greater depth and sheds new light on the taphonomic history of the site’s fossil accumulation. By including all the macro- and meso-vertebrate remains and all the coprolites in our taphonomic analysis, we can provide fresh information on the intervention of biological and natural processes.

## Results

### Archaeological-taphonomy

In this study, we analysed 3640 specimens (NSP). The good state of preservation of these fossil remains meant we were able to determine a high number of them. Indeed, 3608 specimens have been identified at the anatomical and taxonomic levels (NISP = 99.1%). The fossils belong to large and medium-sized ungulates, carnivores, leporids and birds (Table 1). Despite this apparent diversity, the faunal association is composed primarily of the remains of leporids (NISP = 84.1%), lynxes (NISP = 8.2%) and birds (NISP = 6.5%), these three taxonomic groups making up more than 98% of the NISP. Thirty-two specimens were classified according to size, the majority being categorized as small-sized mammals. Based on the NISP, these probably correspond in the main to bone fragments of small carnivores (Table 1). Simpson’s diversity index (D = 0.72) points to the predominance of one taxon (the leporids), while Shannon’s evenness index indicates an uneven distribution of the species represented (E = 0.24).

**Table 1:** NSP, NISP, MNE and MNI and frequencies of the fossil remains recovered in the layer IIIa of the Cova del Gegant.

Taxa	NISP	%NISP	MNE	%MNE	MNI	%MNI
*Equus* cf. *ferus*	4	0.11	3	0.1	1	1.12
Bovidae indet.	1	0.03	1	0.03	1	1.12
*Cervus elaphus*	3	0.08	3	0.1	2	2.25
Ungulate indet.	3	0.08	3	0.1	0	0
Hyaenidae cf. *Crocuta crocuta*	2	0.06	2	0.07	2	2.25
*Canis*/*Cuon* sp.	4	0.11	4	0.1	1	1.12
*Vulpes vulpes*	3	0.08	3	0.1	1	1.12
*Lynx pardinus*	299	8.29	273	9.1	6	6.74
*Felis silvestris*	10	0.28	10	0.3	2	2.25
Carnivora (small)	8	0.22	3	0.1	0	0
Leporidae	3035	84.12	2492	83.4	56	62.9
Aves	235	6.51	189	6.3	16	18
*Emys*/*Testudo*	1	0.03	1	0.03	1	1.12
**Total NISP, MNE, MNI**	**3608**	**100**	**2987**	**100**	**89**	**100**
Large size	1	0.03				
Medium size	8	0.22				
Small size	12	0.33				
Very small size	1	0.03				
Indet.	10	0.27				
**Total NSP**	**3640**	**100**				

The estimate of the assemblage’s MNE is high at 2987, most belonging to the dominant taxa (MNE = 98.9%). Based on these elements, we calculate a minimum number of 89 individuals, distributed as follows leporids: 56, birds: 16 and lynxes: 6, and accounting for 87.64% of the total MNI.

Due to the differences in the respective methodologies employed to study macro-mammals and meso-vertebrates (leporids and birds in this work), we present the data in two different sub-sections.

#### Macro-mammals

The total number of macro-mammals remains (NSP) in our analysis is 358 (337 NISP). This includes all ungulate, carnivore and taxonomically indeterminable remains attributed to weight sizes that are not compatible with animals considered as meso-vertebrates in our study.

The anatomical representation of the macro-mammals in level IIIa of the sequence is heavily biased, with the except of the lynx that shows a high degree of integrity. Cranial elements include isolated teeth of horse and red deer, and a fragment of the horn core of an indeterminate bovid (*Bos*/*Bison*/*Capra*). Postcranial bones include one unfused distal tibia of an indeterminate small ungulate; one complete metacarpal and one capitatum-trapezoid of an adult red deer, probably corresponding to the same individual; one complete patella of an adult horse; and two fragments of trunk elements: the corpus of a thoracic vertebra and one rib fragment from an indeterminate medium-sized ungulate. Among the 32 remains classified by size, nine – in the main trunk specimens – are compatible with that of the ungulates represented. Ten elements have been estimated by association with the ungulate specimens identified, belonging to a minimum of four individuals (Table 1). At least two of these are young individuals (one horse and one deer) and another (deer) is a prime adult.

As in the case of the ungulates, the skeletal completeness of the carnivores is very low. Specimens of large canid (probably *Canis lupus*) and hyena correspond mainly to distal foot bones (tarsal, metapodials and phalanges). These bones correspond to one adult canid and two hyenas – one young and one a prime adult. One red fox adult individual is represented by an extraordinarily well-preserved skull, one fragment of hemi-mandible and one fragment of innominate. Ten specimens (NISP = MNE) from cranial, appendicular and axial post-cranial segments indicate the presence of two adult wild-cat individuals in the assemblage.

**Figure 2:**
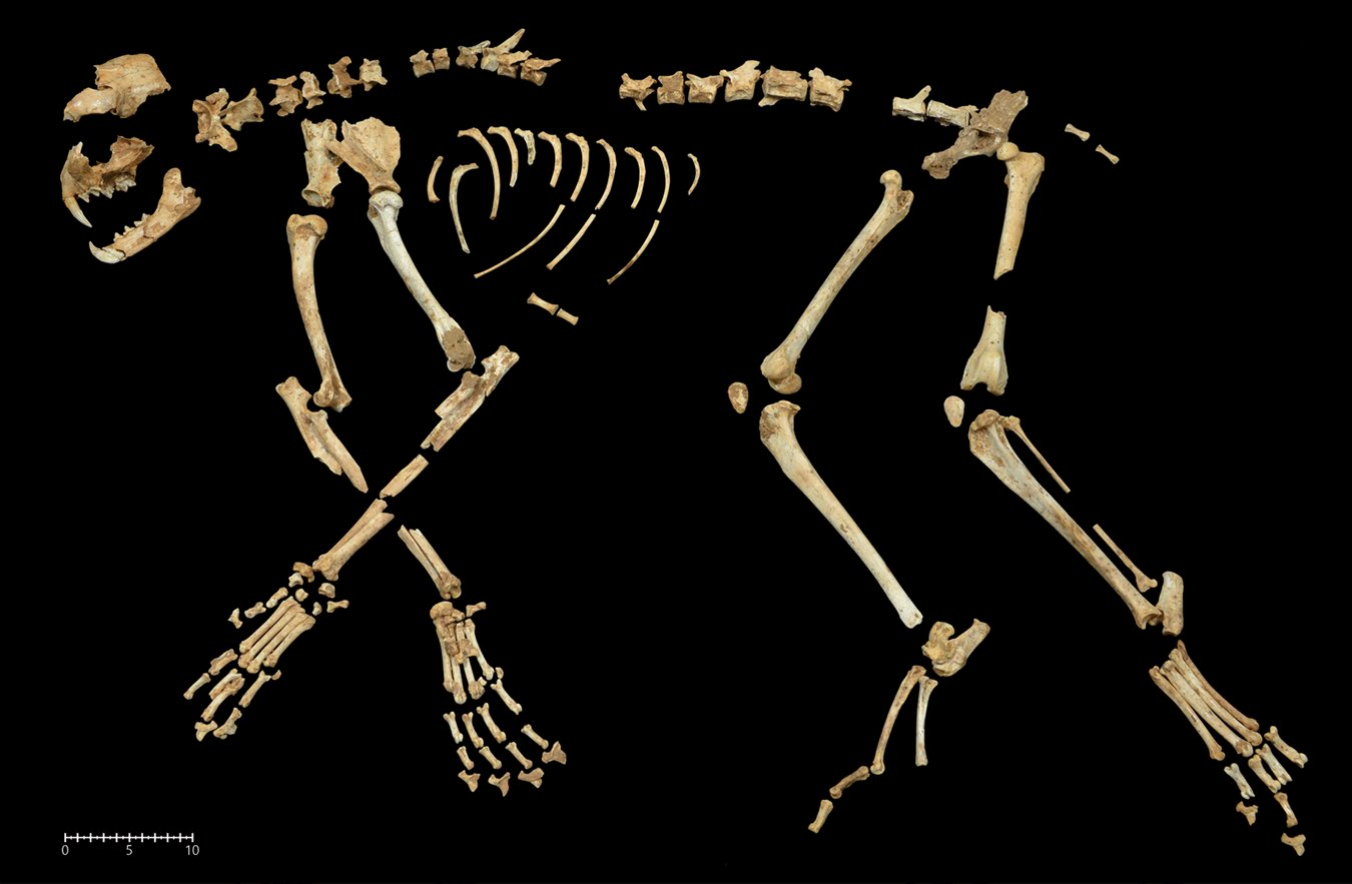
Skeletal reconstruction of Iberian lynx from different individuals. Example of the completeness of skeletal representation of Iberian lynx in the layer IIIa of Cova del Gegant.

The c. 300 lynx specimens merit more detailed attention. The skeletal representation of lynx shows a high degree of completeness (Table 2 and Fig. 2). All skeletal elements are represented, the most abundant in NISP being phalanges, vertebrae, metacarpal and carpal bones. The 299 specimens identified have allowed us to estimate a minimum of 273 elements. Both the most and least represented elements are the same as for the NISP, which again is indicative of the completeness of the lynx skeletons. Based on the different degrees of development of the upper teeth, we estimate a minimum of six individuals. One of them is an infant, just a few weeks old; another a juvenile of less than a year; and four individuals correspond to prime adults (Table 2 and Fig. 2). Most of the specimens correspond to the adults or the young juvenile individual, while the lynx kitten is represented by just a few specimens. In relative terms (%ISU), the anatomical representation of lynxes shows high frequencies for the high-survival elements, including skulls, isolated teeth and the main limbs, and low frequencies for small bones, such as sesamoids and tarsals. Some of the low-survival elements, above all ribs, are poorly represented but vertebrae are relatively well preserved. The mean %ISU is 41, suggesting the survival of almost half the skeletons.

**Table 2:** NISP (%), MNE (%), MNI, %RA and %ISu of the main represented taxa by elements recovered in the layer IIIa.

	Leporidae (MNI 56)					Aves (MNI 16)					*Lynx pardinus* (MNI 6)				
Element	NISP	%NISP	MNE	%MNE	%RA	NISP	%NISP	MNE	%MNE	%RA	NISP	%NISP	MNE	%MNE	%ISU
Cranium	90	3	25	1	45	0	0	0	0	0	7	2.3	6	2.2	100
Mandible	103	3.4	87	3.5	78	0	0	0	0	0	3	1	5	1.8	41.7
Incisor	110	3.6	110	4.4	33	0	0	0	0	0	0	0	0	0	0
Upper molar	150	4.9	150	6	22	0	0	0	0	0	0	0	0	0	0
Lower molar	234	7.7	234	9.4	42	0	0	0	0	0	0	0	0	0	0
Tooth	0	0	0	0	0	0	0	0	0	0	13	4.3	0	0	0
Hyoid	0	0	0	0	0	0	0	0	0	0	3	1	3	1.1	25
Vertebra	206	6.8	109	4.4	5	18	7.7	18	9.5	9.2	40	13.4	36	13.2	21.4
Rib	107	3.5	52	2.1	4	0	0	0	0	0	15	5	15	5.5	9.6
Coracoid	0	0	0	0	0	22	9.4	19	10.1	73.1	0	0	0	0	0
Furcula	0	0	0	0	0	1	0.4	1	0.5	7.7	0	0	0	0	0
Sternum	0	0	0	0	0	1	0.4	1	0.5	7.7	6	2	2	0.7	33.3
Scapula	32	1.1	28	1.1	50	8	3.4	8	4.2	30.8	5	1.7	5	1.8	41.7
Humerus	99	3.3	71	2.8	63	19	8.1	12	6.3	46.2	3	1	3	1.1	25
Radius	83	2.7	57	2.3	51	5	2.1	4	2.1	15.4	6	2	5	1.8	41.7
Ulna	63	2.1	51	2	46	37	16	25	13.2	96.2	9	3	9	3.3	75
Femur	136	4.5	76	3	68	17	7.2	13	6.9	50	13	4.3	9	3.3	75
Patella	10	0.3	10	0.4	9	0	0	0	0	0	3	1	3	1.1	25
Tibia	164	5.4	111	4.5	99	27	11	22	11.6	84.6	6	2	6	2.2	50
Fibula	0	0	0	0	0	0	0	0	0	0	3	1	2	0.7	16.7
Innominate	83	2.7	52	2.1	93	0	0	0	0	0	3	1	3	1.1	25
Synsacrum/Pygostyle	0	0	0	0	0	2	0.9	2	1.1	15.4	0	0	0	0	0
Metacarpus	121	4	111	4.5	20	0	0	0	0	0	23	7.7	23	8.4	38.3
Carpometacarpus	0	0	0	0	0	18	7.7	13	6.9	50	0	0	0	0	0
Metatarsus	375	12.4	289	11.6	65	0	0	0	0	0	11	3.7	11	4	18.3
Tarsometatarsus	0	0	0	0	0	22	9.4	13	6.9	50	0	0	0	0	0
Astragalus	102	3.4	102	4.1	91	0	0	0	0	0	4	1.3	4	1.5	33.3
Calcaneum	36	1.2	36	1.4	32	0	0	0	0	0	7	2.3	7	2.6	58.3
Carpal	0	0	0	0	0	0	0	0	0	0	16	5.4	16	5.9	16.7
Tarsal	0	0	0	0	0	0	0	0	0	0	5	1.7	5	1.8	6
Carpal/Tarsal	51	1.7	51	2	4	0	0	0	0	0	0	0	0	0	0
Digit	0	0	0	0	0	4	1.7	4	2.1	15.4	0	0	0	0	0
Phalange	680	22.4	680	27.3	23	34	14	34	18	8.7	81	27.1	81	29.7	26
Sesamoid	0	0	0	0	0	0	0	0	0	0	14	4.7	14	5.1	7.8
**Total**	**3035**	**100**	**2492**	**100**	**—**	**235**	**100**	**189**	**100**	**—**	**299**	**100**	**273**	**100**	**—**

Fragmentation of the macro-mammal remains is rare with 257 elements remaining complete (69% of MNE), including 24 of the main long bones of lynx (19), wild cat (4), large canid (1), and one red deer metapodial. In the specific case of the lynx, the percentage is higher (79.5%). Considering that the lynx represents 90% of the NISP of macro-mammals, the low rate of breakage of skeletal elements suggests very little post-mortem disturbance of the bones. During fieldwork, several anatomical connections were recorded among the lynx elements, including almost complete right forepaw (Fig. 2). Likewise, the two almost complete skulls and the anatomical refitting implemented among the fragments broken under dry conditions reinforce this observation.

Our analysis of long limb bone breakage included only a small number of specimens (20)–40% correspond to bones that preserve a 1/4 or 1/2 of the length of the diaphysis and the whole section and 60% correspond to small fragments i.e. a 1/4 or less of the length of the diaphysis and less than 1/3 of their section. Only eleven fracture outlines have been analysed. More than 63% present right angles, while 100% present irregular surfaces indicating dry post-depositional breakage. Fractures produced during the excavation process affect 24 specimens; diagenetic breakage 32 and only one fragment has been attributed to fresh (green) breakage generated by carnivores in association with a type A notch. Unfused bones (12) and bones with indeterminate fractures (9) complete the assemblage. According to the data presented, the scarce fracturing of the sub-set of macro-mammals can be said to have occurred when the bones were in a dry/fossilized state.

#### Meso-vertebrates

A total of 3271 specimens (NISP) correspond to mesofauna, primarily leporids and small birds. As noted above, leporids are the most abundant with 3035 identified remains (NISP) (Table 1). Based on left tibia records, a total of 56 individuals (MNI) has been estimated. The mortality pattern shows a predominance of adults > 9 months (MNI 36 or 63%), followed by young individuals (MNI 36%). Among the latter, at least six are perinatal, 13 infantile <3 months and one is juvenile of 3–5 months.

The skeletal elements have all been recovered in different percentages. Phalanges, metatarsus, isolated lower molars and vertebrae are the best represented anatomical elements in absolute terms, while patellae, scapulae, astragals and articular bones appear in smaller numbers. The MNE is 2492, dominated by phalanges (Table 2). The relative abundance (%RA) of the skeletons presents a predominance of tibiae, innominate, calcaneus, mandibles, femurs and metatarsus (up to 60% of the RA) and a lower presence of ribs, vertebrae, articular bones (carpal/tarsal) and metacarpal (less than 10% of the RA). The best represented element in relative terms is the tibia (Table 2).

The relative proportions of skeletal remains indicate a greater representation of the postcranial than of the cranial skeleton (Supplementary Table S1). However, if we evaluate the accuracy of this test, the evenness of proportions is less pronounced. Thus, the TA/MD index that considers two elements, ranks mineral density highly^45^, and reveals higher valance proportions in the cranial/postcranial representation (159.2), especially if we consider the MNE (TAE/MDE = 127.5). In the case of limb bones, the lower limbs (metapodials) are better represented than the intermediate limbs (250.4), which, in turn, are better represented than the upper limb bones (131.9). Finally, the forelimb is slightly better represented than the hindlimb (54.2) in the Cova del Gegant assemblage. This relationship becomes more pronounced as we proceed from proximal to distal elements (Supplementary Table S1).

A study of bone breakage shows that 55.6% of the skeletal remains of leporids are complete. However, there is no statistically significant correlation between the size of the complete element in comparative specimens and their completeness in the Cova del Gegant assemblage (rho = 0.1, p > 0.05). Yet, the largest elements in a complete skeleton, including the main long bones, skull and girdles, are usually broken while the shortest, including phalanges, metacarpal and articular bones, are usually complete. No complete ulnae, scapulae or ribs have been recovered. The size of the leporid remains ranges between 1.2 and 93.56 mm (mean = 16.03 mm), with more than 61% being greater than 10 mm in length. In the case of limb bones, the upper and intermediate (UILB) bones are highly fragmented with only 43 complete bones (7.8% of the UILB), represented primarily by femurs (39.53% of the complete UILB) and humerus (30.23% of the complete UILB), while the lower limbs (metacarpal and metatarsal) are mainly complete (71 and 53%, respectively). The fragmentation of long bones indicates the same frequencies for proximal (PE + PES) and distal (SDE + DE) parts (~30%). The breakage categories included in Supplementary Table S2 show that the cranium is represented mainly by NC and M fragments and the mandibles by MBI. Complete elements in skulls are more abundant among mandibles (9.71%) than they are in crania (1%). Innominate are represented by fragments that retain the acetabulum (AIS, AISIL, AIL), while scapulae are represented by GCN fragments. Ribs are mainly shaft (S) or shaft retaining epiphyseal fragments (PES). Articular bones, *in situ* teeth and isolated teeth are represented mainly by complete elements (Supplementary Table S2). Some anatomical connections have been recorded, such as tarsal with metatarsal bones and carpal with metacarpal elements.

**Figure 3:**
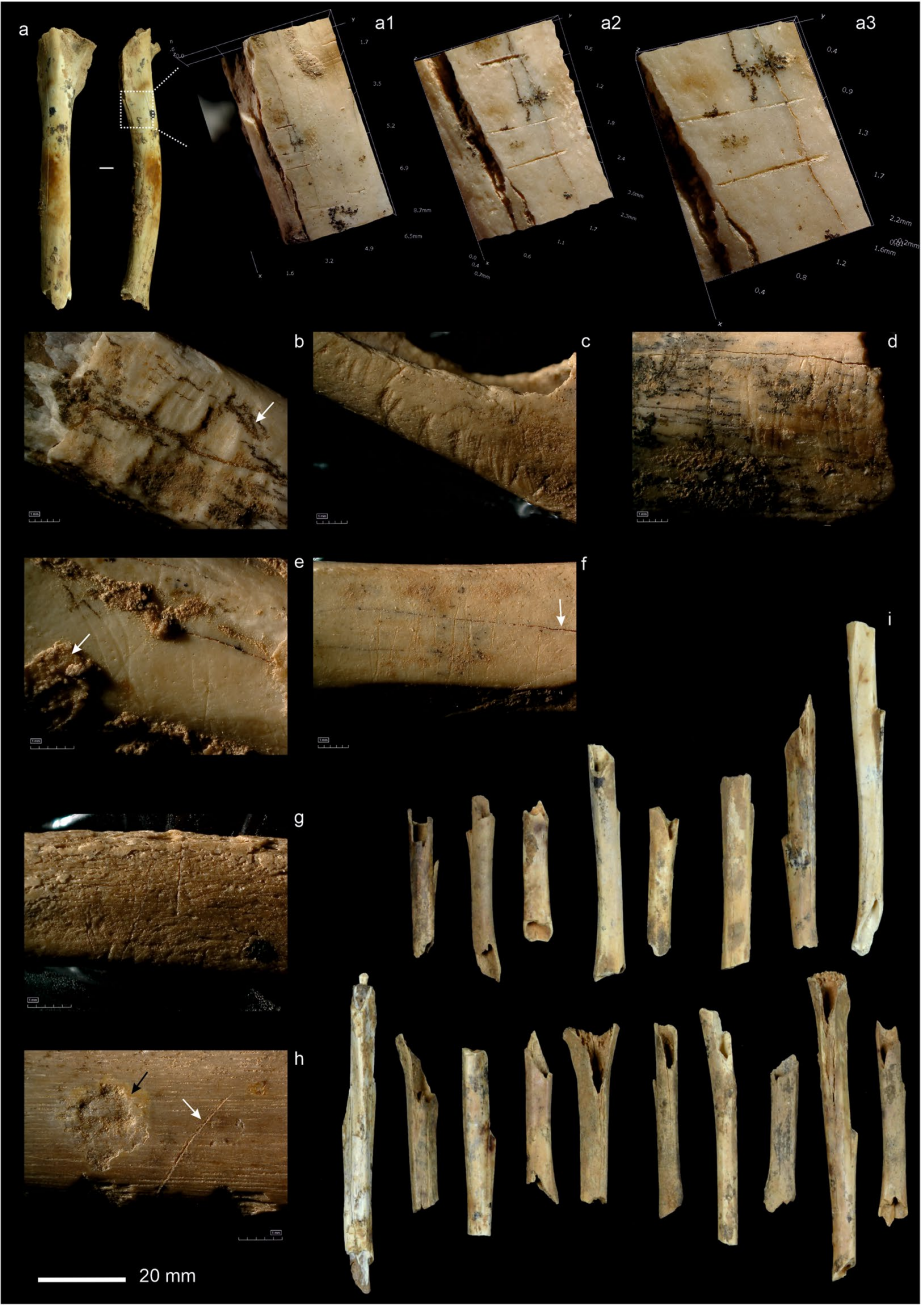
Bone surface modifications attributed to post-depositional agencies. (**a**) Trampling marks at different magnifications, (a1) x35, (a2) x100, (a3) x140, (**b**,**c**), rodent gnawing, (**d**) scratching, (**e**) scratching and cementations (white arrow), (**f**) scratching (white arrow indicates fissures), (**g**) trampling in bird scapula, (**h**) biochemical alteration (black arrow) and trampling (white arrow), (**i**) fake-tubes. Scale bar is 20 mm (in A4, 1:1).

The bone breakage study revealed a mixture of green and dry fractures with a predominance of the former. Sixty-two rich-marrow long limb bones (humerus, femur and tibia) retain the epiphysis plus a fragment of diaphysis presenting a helical or V-shaped fracture that can be classified as morphotypes of carnivore breakage^20,29,45^. They include 19 distal humerus, 27 distal tibiae and 2 distal and 14 proximal femurs. Although in Supplementary Table S1, 86 remains are classified as shaft (S), only the main long bones (humerus, femur and tibia) contain enough features to establish the state (dry or green) in which the fracture occurred. Thus, only one diaphyseal cylinder presenting green fractures (tubes) has been observed, although at least 30 remains display midshaft cylinder morphology (fake tubes) (Fig. 3i). All the evidence points therefore to a high incidence of diagenetic and recent (excavation-storing) breakage.

Avian remains comprise the third most abundant taxonomic group in the Cova del Gegant IIIa assemblage. Of the 235 remains, 127 (54.04%) have been identified taxonomically. The rest have been identified anatomically and categorised as small birds. The specimens identified correspond to *Pyrrhocorax graculus* – the yellow-billed chough (NISP 61, MNE 50, MNI 7), *Pyrrhocorax pyrrhocorax* – the red-billed chough (NISP 61, MNE 45, MNI 6), *Columba livia*/*oenas* – the rock/stock dove (NISP 2, MNE 2, MNI 1) and *Alectoris* cf. *rufa* – the red-legged partridge (NISP 2, MNE 2, MNI 1). One piece of proximal tarsometatarsal bone from *Accipiter gentilis* – the northern goshawk, represents the only medium-sized bird specimen.

We estimated 189 of the elements, 31 of which correspond to young corvid individuals (13.19% of the NISP). The ulnae and tibiae indicate the presence of a minimum of 13 corvids (MNI). These, together with the remaining three individuals give a total of 16 MNI for birds in the assemblage. All anatomical elements are represented except for the cranium and ribs. The most represented in relative terms (%RA) are the ulna, tibia and coracoid, while the furcula, sternum and vertebrae are the least represented (Table 2). Relative proportions of skeletal elements and portions are shown in Supplementary Table S1. Our results indicate a degree of balance between wing and leg elements and between proximal and distal elements, while those of the limbs are more abundant than those of the core.

Breakage is high among the bird remains, especially in the case of limb bones that preserve proximal (38.9%) or distal parts (36.6%). A total of 90 elements are complete, of which 51 are complete phalanges (mainly pedal) and cervical vertebrae. In six cases, limb bones are represented by shaft fragments. However, as with the leporids, these shafts present features indicative of dry/post-depositional breakage (Supplementary Table S3). All the limb fragments analysed show transversal delineation in their fracture planes and shatter surfaces (97.3%). The few fracture planes that present smooth surfaces (3.7%) display right angles. This combination of features indicates dry/post-depositional breakage as the norm in the avian assemblage in layer IIIa of Cova del Gegant.

#### Bone surface modifications (BSMs)

The cortical surface of the bones in the assemblage is very good allowing us to present the percentage of modifications as a proportion of total remains (NSP). The most abundant modifications in terms of frequency, for both the macro- and meso-fauna, are those of geological/post-depositional origin (Supplementary Table S4). These modifications affect all the remains with a similar frequency and intensity, regardless of whether they are macro- or meso-vertebrate remains. Biological (mainly carnivore) modifications in contrast differ in their occurrence between macro-mammals, leporids and birds, although the frequency is similar (see below).

The modifications associated with the karstic environment, i.e. concretions, manganese coating, humic staining and humidity-related surface alterations such as exfoliations and fissures, are the most frequently documented. Manganese coating affects 27% of the remains in the assemblage, while root and organic staining affects 10.4%. Exfoliation, cracking and fissures resulting from changes in humidity (referred to here as degree 1 weathering) affect 9.4% of the specimens, the same proportion than concretions (CaCO_3_ cementations). Deformations due to sediment pressure affect only five macro-mammal remains. Classic trampling marks, identified as groups of striae, straight grooves and parallel organization, are recorded in four macro-mammal and in five avian remains, while groups of scratches of undetermined origin (albeit like trampling marks) affect seven leporid remains. Of the leporid specimens, just 0.2% present concentrations of very shallow grooves like trampling marks, referred to here as *scratchings* (Fig. 3). Classic trampling affects 1.1% of the macro-mammal remains and 2.1% of the bird bones. Biochemical modifications on bone surfaces in the form of irregular grooves and pits with corrosion around the borders and dissolution in the form of rounded cupules with rounded edges and a curved bottom are rare, affecting just 1% of NSP. Rodent gnawing was recorded on seven leporid remains.

In addition, we have observed modifications generated by carnivores in the form of tooth marks, digestion marks and carnivore breakage. Macro-mammal remains were only affected by carnivore modifications in two cases (0.5%). One shaft fragment from an indeterminate long bone (small mammal) presents a type A notch associated with tooth pits; however, the moderate digestion of the fragment prevents us from taking the pit measurements. Furthermore, a corpus fragment of an immature ungulate vertebra presents a score that is 1.07 mm in width

**Figure 4:**
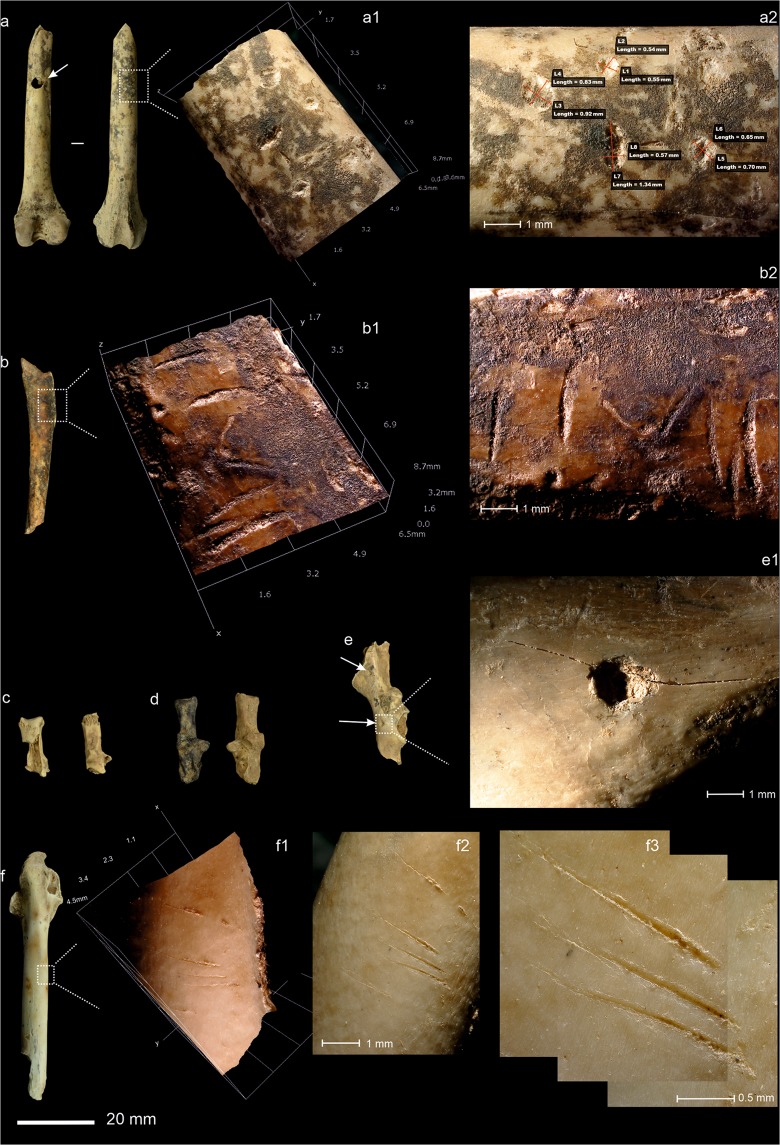
Bone surface modifications attributed to biological agencies. (**a**) Carnivore tooth marks, puncture (white arrow), and pits at different magnifications, (a1) x35, (a2) measurements by HIROX KH-8700 3D software, (**b**) carnivore scoring, (b1-b2) at different magnifications x35, (**c**) digested bones, (**d**) burning damage (left) vs. non-burned leporid calcaneus, (**e**) carnivore pits in innominate, (e1) and detail at x35 magnifications, (**f**) cut marks in femur of leporid at different magnifications, (f1) x35, (f2) x50, (f3) x140. Scale bar is 20 mm (in A4, 1:1).

Forty leporid specimens display tooth marks in the form of pits (14 NISP), punctures (16 NISP) and scores (10 NISP). This, however, is a very small fraction of the assemblage of leporid bones (1.32% of the NISP). The marks appear mainly on innominate (32.5%), humerus (20%), femur and tibia (12.5%). In absolute terms, the most frequently gnawed element is the innominate and humerus (15.66 and 8.08% of NISP, respectively). In the case of the long limb bones, tooth marks mainly appeared on shaft portions and were normally isolated pits, although punctures appeared on fracture edges (4 NISP) and opposite pits/punctures (6 NISP) too. The tooth marks on innominate are mainly punctures (Supplementary Table S5 and Fig. 4). The size of the pits in the cortical tissue (shaft of long bones) point to tooth marks that are slightly larger than the experimental data recovered for both lynxes and foxes on leporid carcasses (breadth mean of 1.67 mm vs. 1.38 mm and 1,62 mm recorded in experimental works conducted with Iberian lynx on rabbit carcasses^20,29^ (Supplementary Fig. S1)). In the case of the scores, we documented nine bones with multiple small scores similar to those made by lynx cubs observed in experimental studies^29^ (Fig. 4b). Among the carnivore modifications, note should be made of the signals of digestion affecting 40 leporid remains, mainly on humerus (50%), phalanges (15%), and metatarsus (7.5%). In relative terms, the elements of the hindlimbs, especially the humerus (20.2% of the humerus NISP) and scapula (6.25% of scapula NISP), were affected. The degrees of digestion were mainly light (48.9%) and moderate (40.4%), and heavy in just 10.6% of the remains (Fig. 4). No single leporid bone presents extreme digestion damage.

Anthropogenic modifications of bone are largely testimonial but, nevertheless, present (Fig. 4). The most frequent are thermo-alterations (58 NSP or 1.6%), associated, it can be presumed, with the combustion structures documented previously in layer IIIa^44^. This burning has affected the main taxa (lynx, birds and especially leporids) and some indeterminate remains to different degrees, ranging from rubefaction (grades 1/2 = 3) and cremation (grade 4/5 = 40) to calcination (grade 6 = 5). Butchering marks are totally absent except for five slicing marks located on the midshaft of the femur of an adult leporid. The cuts are clustered in the proximal meta-diaphysis on the anterior to medial side. All the slices show straight delineation, oblique orientation and a parallel correlation. The cuts are presumably the result of defleshing (Fig. 4f), while the femur has lost its distal epiphysis as the result of a modern fracture. During the analysis undertaken here and during the process of reviewing the manuscript, we (and 2 reviewers) have called into question the diagnosis of these marks more than once. Indeed, the assemblage contains other marks that are not readily diagnosed (see Figs. 3a and 4f), but which are presumably the result of geological processes, such as trampling. Observed at a lower magnification (e.g. hand lens), or under oblique light, these marks can easily be interpreted as cut marks. However, these difficulties, once again^46^, highlight the challenge of diagnosing certain taphonomic marks and stress the need to use recently developed methods (e.g^47^.) to solve equifinality problems. The fact that we have found a single remain with what appear to be cuts, but on which other anthropogenic marks of butchering are absent, means we have to be cautious in our interpretation of these marks. However, two conclusion can be drawn: i) these putative cut marks can be related to the ephemeral activities conducted in the cave in relation to fireplaces and ii) the scarce or non-existent human modifications of the bones analysed here suggest a fairly homogeneous assemblage and one that is, therefore, optimal for analysing lynx signatures.

**Figure 5:**
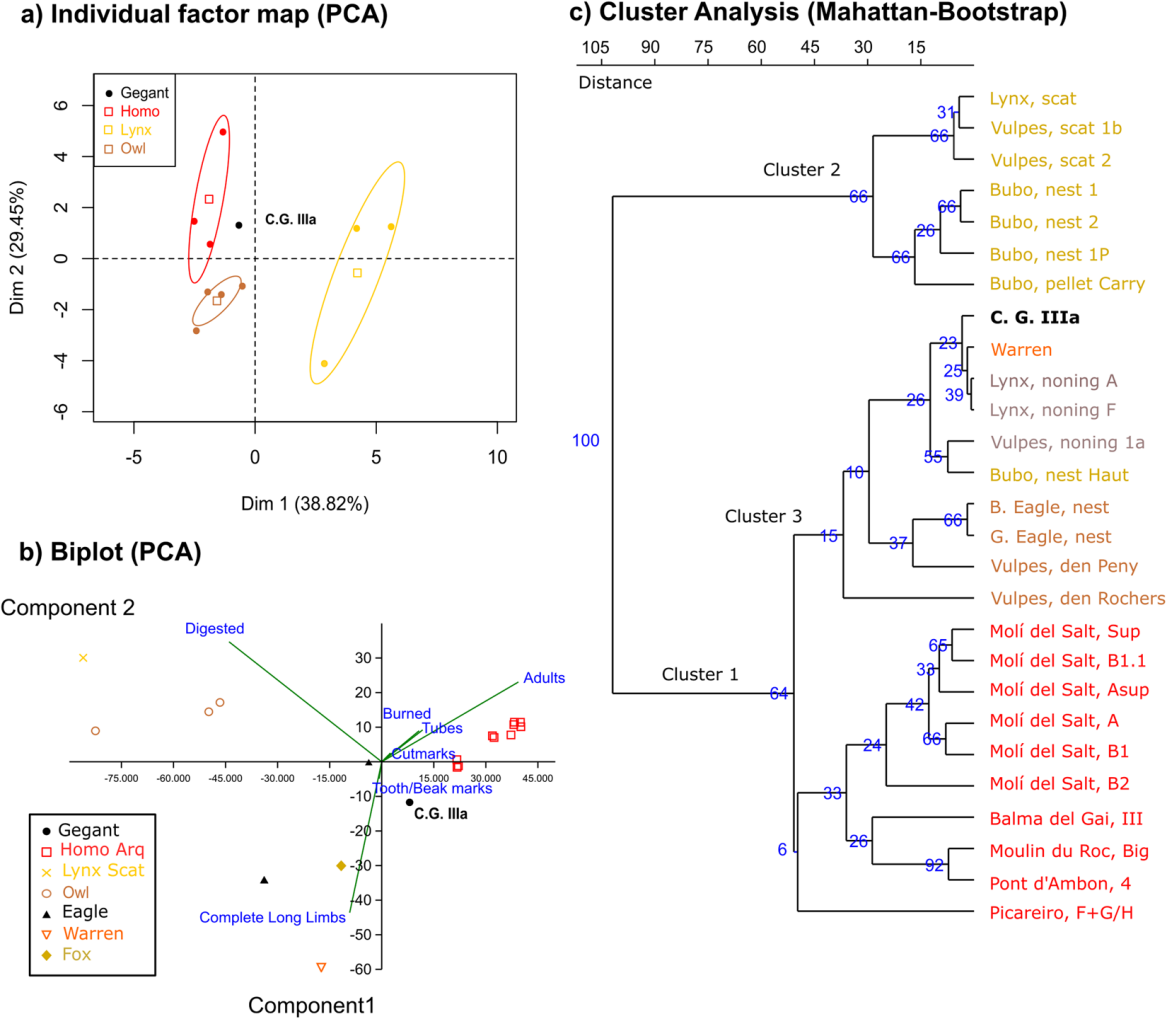
Comparative multivariate analysis of taphonomic features of layer IIIa at Cova del Gegant. (**a**) Principal component analysis of anatomical representation of leporid remains in several experimental and archaeological assemblages, (**b**) Principal component analysis of several taphonomic attributes of leporid assemblages generated by different taphonomic agents and processes, (**c**) Cluster analysis of several taphonomic attributes of leporid assemblages generated by different taphonomic agents and processes. To see reference data Supplementary Tables S6 to S8.

The comparative multivariate analysis conducted between the leporid assemblage in layer IIIa of Cova del Gegant and several actualistic and archaeological assemblages of reference was based on two principal component analyses. The first examined the anatomical composition in terms of minimal animal units (%MAU) of the leporid remains in the assemblages (Fig. 5a) drawing on the data in Supplementary Table S6. The second combined taphonomic (% cut marks, % burned bones, % digested bones and % tooth/beak marks) with breakage data (% complete long limb bones) and mortality (% adults) (Supplementary Table S7) (Fig. 5b). The cluster analysis uses five taphonomic criteria (% cut marks, % burned, % digested, % tooth marks and % midshaft cylinders or tubes) (Supplementary Table S8). Results indicate the influence of two main taphonomic agencies, terrestrial carnivores, probably the lynx, and natural depositions in the assemblage of Cova del Gegant (Fig. 5c).

#### Coprolites

Two different morphotypes can be identified in layer IIIa at Cova del Gegant. However, morphotype 1 is represented by a single fragment, making morphotype 2 by far the most abundant (N = 147). Coprolites are dominated by shapeless specimens (N = 131. 89%), followed by fragments (N = 15.1%) and, finally, complete coprolites (N = 2.1%).

Their internal structure – comprising a spiral fabric and friable hardness – is the most frequently occurring feature allowing us to ascribe the vast majority of coprolites to morphotype 2. No aggregates are observed by the naked eye in the interior, which are spongy and present voids.

The morphometry of five coprolites (morphotype 2) was analysed. Morphotype 2 ranges in length from 17 to 50.46 mm and from 17.71 to 23.68 mm in diameter. The latter being the most significant dimension for discriminating between carnivore faeces. Cova del Gegant coprolites are clearly distinct from those of the hyena (morphotype 1) and the scats of the large felids (morphotype 2), including the mountain lion (*Puma concolor*) and jaguar (*Panthera onca*) (Supplementary Fig. S2). The five specimens are similar in size to the scats of the modern coyote (*Canis latrans*, mean of 21 ± 0.2 mm), wolf (*Canis lupus lupus*, mean of 19.70 ± 2.45 mm) and lynx (*Lynx pardinus*, mean of 21.8 mm). However, the scats of other small carnivores, such as those of the red fox (*Vulpes vulpes*, mean of 14 ± 0.2 mm) could overlap, given that their range is between 8 and 20 mm^48^.

Bone and tooth content are abundant in most of the coprolites (Supplementary Table S9). The bones are dominated by leporid remains (NR = 46.66%), followed by small mammal remains (NR = 7.1%) and shaft fragments from leporid or small mammals (NR = 9.13%). Larger mammals are represented by just five remains (6%). The four ulnae identified are represented by the proximal portion and the radius by the distal part.

## Discussion

Several of the features detected in the assemblage point to the presence of a lynx den in layer IIIa of Cova del Gegant. Based on the biostratinomic alterations, our results strongly indicate that the Iberian lynx was the main bone modifier and accumulator of the fossil assemblage. While the assemblage is undoubtedly an accumulation of mixed origin, in which different agents and processes have intervened in the formation of the deposit, layer IIIa allows us to characterize it as the taphocoenosis of the Iberian lynx. Moreover, the BSMs generated by fossildiagenesis are abundant and have played a key role in the final configuration of the layer IIIa assemblage. However, in this sense, the data presented here contribute to support previous interpretations^44^.

Most of the taphonomic modifications recorded in layer IIIa are related to the feeding behaviour of mammalian carnivores. Tooth marks and digestion corrosion are especially prominent in the bones of the rabbit, the main prey here of the taphonomic accumulator. The frequency of the tooth marks, their location, the number of marks per element, the morphology and dimensions of the pits on the rabbit bones are all compatible with the neo-taphonomic record of the Iberian lynx^18,20^. While it is true that the dimensions of the tooth pits on the cortical tissue are slightly larger than those recorded in actualistic studies of current Iberian lynx^20,29^, recall that *L. pardinus* comes from a long anagenetic lineage (*L. issidorensis- L. pardinus spelaeus- L. pardinus pardinus*) characterized by the progressive reduction in body size^49^. This reduction in body size of the post-glacial lynx could explain the differences observed between the fossil assemblage and current records. Likewise, the number of pits is very low, and the methods employed appear to have changed, and despite the comparative analyses conducted here to illustrate our results, with a sample of fewer than 30 tooth pits, our statistical results are not fully representative. Nevertheless, even when we take into account the differences in tooth pit size and the differences in the methods employed, our results still indicate a considerable overlap between the means (95% CI) of the breadth of the tooth pits in the cortical bone found in experimental studies conducted with Iberian lynx and those found in our data from layer IIIa at Cova del Gegant. As such, these dimensions could be used as a reference for the tooth marks of medium-sized felines in archaeo-paleontological assemblages.

Moreover, small tooth scoring marks, associated with the presence of lynx cubs^29^, have been recorded too. Based on actualistic data, the gnawing damage caused by the fox on rabbit bones is very similar in terms of location, morphology and size of the pits to that generated by the Iberian lynx^e.g.^ ^24^. Having said that, tooth marks are slightly more frequent in the case of canids and the occurrence of multiple pits/scores per element is rare in assemblages modified by felids yet common in those modified by foxes^20,24,25,29,32,50–52^.

Modifications generated by large raptors, such as beak/talon marks, and complete or near-complete long bones presenting marks of digestion are totally absent in our assemblage, which allows us to rule them out as modifying agents.

Digested bones are extremely scarce here compared with the high percentages recorded in actualistic studies for different leporid accumulators, especially compared to the high frequencies reported for nocturnal and diurnal raptors^5,16,17,19,21,23,27,28,31,53–55^. However, the analysis of the scats of small carnivores, such as lynx and fox, indicates high proportions of digested remains, especially those presenting heavy or extreme damage^18,24,26,32^. Taking into account the large presence of coprolites and fragments of coprolites in layer IIIa, the scarce presence of digested bones, and the total absence of extreme grades of digestion, may be related to the differential destruction of poorly preserved bones in archaeological sites^56^; however, our own errors in identifying the correct degree of digestion cannot be entirely ruled out here^57^. The morphology, dimensions and content of the coprolites associated with the faunal assemblage all point to the cave having served as a den for Iberian lynx. When denning, low mobility cubs tend to defecate inside the den, and it is the mothers, as in other fields, that are responsible for burying and removing droppings to avoid attracting the attention of predators. This has been detected before in the archeo-paleontological record of at least two sites with Iberian lynx accumulations^56^.

The variety of species represented and their frequency allows us to discard the large carnivores as the main bone accumulators of the assemblage^58–63^ and points rather to medium-small predators. The taxonomic representation is fully compatible with the diet of the Iberian lynx^2,64^. However, in Mediterranean ecosystems, both small carnivorous mammals and several nocturnal and diurnal raptors base their diet on rabbits^10,64^. In addition, in this region during the terminal Pleistocene, humans generated accumulations in which rabbits usually represented more than 90% of the remains^7,8^. For this reason, the taxonomic composition of the assemblage has only relative utility for the reconstruction of the taphonomic history.

The notable skeletal completeness of the lynx, and the abundance of remains reinforces its role as an accumulating agent^65–68^. In addition, the anatomical connections are compatible with the *in situ* deaths of individuals^69^ while the presence of infantile and young individuals is common in natal dens^70–72^. Both in our study and in other accumulations attributed totally or partially to the Iberian lynx^38,56,73^, the abundance of adults is frequent and difficult to explain. In other nearby Pleistocene sites, where lynxes were also involved in the accumulations, some lynx bones present clear evidence of carnivore damage^74,75^, but this is not the case at Cova del Gegant. Thus, the parsimonious explanation at our site would appear to be that the remains are associated with the natural death of adult individuals.

We cannot rule out the possibility that some elements – in particular, those of the ungulates – might have been sporadically introduced by large carnivores. In fact, the presence of at least one coprolite of morphotype 1 and some large bones with digestion corrosion are compatible with the contribution of hyenas to the accumulation^59^. However, the predominant contribution by the Iberian lynx is also quite compatible with the occasional introduction of elements by other carnivores. Finally, we cannot rule out altogether the natural origin of the few ungulate remains and the probable intrusion of some of the rabbit and chough remains, as the high frequency of complete long bones, the low incidence of BSMs and the high representation of immature individuals indicate^15,76–78^. For the same reason, the breaking patterns and anatomical composition of the meso-vertebrates are difficult to interpret. Although only a small fraction of the meaty, marrow-rich bones present breaking patterns identical to those observed in actualistic assemblages modified by small terrestrial carnivores^20,24,25,79^, most of them are complete-intact bones or bones fragmented by diagenesis. Moreover, the incidence of natural intrusions distorts taphonomic patterns by mixing an assemblage modified by carnivores with rabbits and corvids that have died naturally, thus invalidating the use of certain proxies, such as anatomical representation, breakage and mortality patterns, as our multivariate analysis has demonstrated. Moreover, the presence of burned bones indicates a secondary input of humans as biostratinomic agents, albeit our analysis of five robust taphonomic criteria rule out any major human contribution.

Apart from layer IIIa of the Cova del Gegant, few other Iberian Peninsula assemblages have previously been interpreted as accumulations generated almost exclusively by Iberian lynx. Two other cases, however, stand out, in which the main taxon is the rabbit (*Oryctolagus cuniculus*): Canyars (layer MLU)^56^ on the Mediterranean coast and the Navalmaíllo Rock Shelter (Layer F)^80^ on the Iberian plateau. A third case is provided by Buraca Escura (layer 2) on the Iberian Atlantic coast, which has been proposed as a lynx-modified accumulation of small-sized ungulates, mainly young ibex (*Capra* aff. *pyrenaica*), in which rabbits are scarce^81^. Here, the cave-dwelling ibex could have been the prey of lynx. Lynx remains are also very abundant in terms of NISP and MNI at the Canyars and Buraca Escura sites, but totally absent from Navalmaíllo. The Iberian lynx can be recognised as a species in the Iberian record for more than a million years^82^ and scholars believe that, in all probability, the origin of the species and the morphological changes observed during its anagenetic evolution are closely related to its highly specialized^82–85^. The trophic behaviour we have observed in Cova del Gegant, and which has also been recorded at the Canyars and Navalmaíllo sites, corroborate this point. However, local variations can be identified due, it seems, to changes in ecosystems, which may have obliged the lynx to prey on other animals, the case of Buraca Escura. More work is needed in this regard since we have no experimental references on the damage caused by Iberian lynx on ungulate carcasses, nor on other prey other than rabbit (except for partridges)^51^.

The Iberian lynx is the most frequently represented carnivore in the Iberian fossil record^86–88^. Their Pleistocene and early Holocene range extends into present-day southern France and the north of the Italic peninsula^89^, regions in which it was probably also abundant in the past. Their fossils are located above all in caves and rock shelters, dating back in its current form at least 1.6–1.7 million years or as *Lynx pardinus spelaeus*, an ancestor already specialized in predation on rabbits^82^. All in all, five conditions make the Iberian lynx an ideal candidate for generating large bone accumulations. These can be taphonomically identified as the following: (i) the Iberian lynx use caves and crevices as reproductive dens, (ii) they systematically transport their prey to these shelters to feed their cubs, (iii) they defecate in latrines, (iv) they are highly specialised predators and their diet is very narrow, and, (v), in common with other carnivores, the Iberian lynx is a great bone modifier and their taphonomic signal is readily tracked.

Based on the results presented in this study, the taphocoenosis of the Iberian lynx can be characterized by:

1. the prevalence of rabbits in the assemblages, followed by other prey such as birds;
2. the high representation of lynx remains in NISP, MNE and MNI, and of both adults and juveniles (cubs included);
3. the presence of the taphonomic signal of the Iberian lynx on rabbit remains, characterized by the greater representation of the posterior appendicular skeleton (especially distal parts), the high breakage of long bones and relative shortage of tooth marks;
4. The small size of the tooth marks (pits and punctures), usually confined to bones from natal dens, and the presence of tooth scoring on long bones attributed to kitten chewing; and
5. The abundance of coprolites (morphotype 2) as a source of variable quantities of digested bones presenting high degrees of digestion but a relative absence of extreme degrees.

## Methods

### Excavation methodology

The current assemblage is the result of fieldwork undertaken by the *Grup de Recerca del Quaternari* (GRQ-SERP, University of Barcelona). In 2007, GRQ renewed fieldwork at the site, performed chronometric dating, established the stratigraphic framework and reanalysed the site’s archaeological record. The current phase in excavations is focused on the back of the main gallery and covers an area of ~7 m^2^. The location of all the remains excavated (including coprolites) was recorded in three-dimensions, according to the 1-m^2^ grid system, and identified by letter and number. Remains were mapped *in situ* prior to removal, whereas unidentified small fragments (<2 cm), postcranial bones of leporids, birds and small vertebrates were bagged by 1-m^2^ units of provenience. The sediments were water-screened using superimposed 5.2- and 0.5-mm mesh screens. The layer was affected by cross cuttings of rodent burrows and storage pits. Thus, burrow trails of fossorial animals were observed at the time of excavation, and the sediment contained in these burrows was excavated separately from that of layer IIIa to avoid bioturbated elements. The archaeological materials analysed here included all the remains recovered during field seasons 2007–2017, including the materials previously described^44^ and the materials between grid rows 22 and 28.

### Archaeological taphonomy

A total of 3640 fossil faunal remains was studied from layer IIIa. The zooarchaeological analysis involved the identification of animal bones to genus or species level (where possible). Their anatomical region of origin (cranial, axial and appendicular) was determined, along with the skeletal element, the portion of that element, side and age (perinatal, juvenile, adult or senile) following standard zooarchaeological methods^90^. The taxonomic composition comprises mainly macro-mammals, leporids and small birds and, so, on occasion specific methods have been used. The number of specimens (NSP), number of identified specimens (NISP), minimum number of elements (MNE), minimum number of individuals^69,91,92^, skeletal survivorship in the form of relative abundance (%RA) for leporids and birds^93^ and the index of survival for macro-mammals (%ISu)^63^ were estimated. To calculate the MNI, we considered complete dental series and isolated teeth, taking into account their grade of eruption and wear^94–96^ and skeletal development patterns, fusion and ossification in line with Barone^97^. The state of development and fusion of the skeletal elements and dental eruption and replacement for lynx were estimated using a combination of several studies^98,99^. The epiphysis fusion pattern is the most commonly used method for establishing the age of rabbits at death. At about 9 or 10 months of age, all the epiphyses of the long bones are fused^26,100–103^. The distal epiphysis of the humerus begins to fuse at about 2 months of age. Therefore, the relative presence of the distal epiphyses of unfused humerus helps to identify the proportion of unweaned rabbits in the assemblages. The distal tibia begins this process at about 3 months. Therefore, the proportion of unfused distal tibiae reflects the proportion of very young individuals, although it is less precise than the identification of unweaned individuals by means of unfused distal humeri^103^.

Following Stiner^104^, we clustered the estimated individuals into three age groups: young, prime-adults and old individuals. To these three age classes, we incorporate perinatal when we note the absence of epiphysis, scarce ossification and very small elements. In order to include the non-identified remains with the identified specimens, we established four weight categories: large (>300 kg), medium (100–300 kg), small (10–100 kg) and very small (<10 kg).

The proportions of leporid skeletal elements were also evaluated using several indices:^17,18^ postcranial in relation to cranial (PCRT/CR, PCRAP/CR, PCRLB/CR, TA/MD, TAE/MDE, HU + FM/CR + MD), loss of distal limb elements (AUT/ZE, Z/E) and the ratio of forelimb to hindlimb elements (AN/PO, HU/FM, RDU/TA, MCP/MTT). In the case of birds, the ratios used were: the representation of wings versus legs (W/L), relation between proximal and distal parts of elements (P/D) and the proportion of core to limb elements (CO/LB) all expressed as a percentage^21,51,105,106^ (see Supplementary Table S1 for abbreviations). We calculated both Simpson and Shannon’s indices to assess taxonomic diversity following the recommendations of Grayson and Delpech^107^.

Breakage patterns were described in terms of the maximum length of all identified skeletal elements. Percentages were calculated for complete remains and isolated teeth. For immature individuals, the diaphysis of long bones with unfused epiphyses were considered complete elements. Breakage categories used for leporids and birds are fully described and illustrated in Lloveras *et al*. ^17^^, see Fig. 1,^^106,108^. Different “breakage categories were used depending on bone type: patellae, carpals and tarsals were classified as complete or fragmented; phalanges were recorded as complete, proximal, distal or fragmented, vertebrae were recorded as complete, vertebral body, vertebral epiphysis or spinous processes; breakage of teeth was calculated separately for isolated and *in situ* elements and they were classified as complete or fragmented”^20^^, p.3030^. Long bone cylinders also called “tubes” in the literature (fragments of long bones with snapped ends resulting from consumption), and V-shaped and helical fractures^109^ were also recorded. In this study, in the case of leporids, we only consider the elements containing an appreciable amount of marrow as humerus, tibia, and femur^4^. We considered as “fake tubes”, the long bone cylinders with sign of dry breakage (jagged or right-angle-smooth ends). Following Bunn^110^ and Villa and Mahieu^109^, we also recorded shaft circumference and shaft length along with fracture outline, angle and edge to explore the nature of fragmentation observed in the assemblage. Notches considered as “semi-circular- to arcuate-shaped indentations on fracture edges with corresponding negative flake scars on medullary surfaces [of limb bones]”^111^^, p. 724^) were also identified and analysed according to the typological classifications proposed by Egeland^112^, although we only recorded one notch (“complete or A type” defined by its having two inflection points on the cortical surface and a non-overlapping negative flake scar).

All skeletal remains were examined both macro- and microscopically. For microscopic observations, a binocular lens with variable magnifications (6.5 to 40) with an oblique cold light source was used. Punctures, pits, scores, crushing and helical fractures, crenulated edges and digestion marks^57,113–115^ were identified and documented. Punctures and pits were also classified by their number (isolated or multiple) and distribution (unilateral or bilateral)^116^. We also noted the size measurements of the pits, punctures and scores using the criteria outlined by Andrés *et al*.^117^ and compared them with experimental data from Andrés *et al*.^117^, Massigogue *et al*.^52^,and Rodríguez-Hidalgo *et al*.^20,29^. Cut marks were identified on the basis of the criteria outlined by Potts and Shipman^118^, Shipman and Rose^119^ and Domínguez-Rodrigo *et al*.^120^. Damage attributable to burning was described by colour (naked eye) using the six-grade scale proposed by Stiner *et al*.^121^: (1) slightly burned, (2)>half carbonized, (3) fully carbonized, (4) slightly calcined, (5)>half calcined and (6) fully calcined (completely white). Other modifications associated with the depositional history of the bone assemblage such as the presence of roots etching, chemical corrosion, biochemical marks, manganese oxide staining and trampling have been identified following the criteria detailed in several works^122–124^. Detailed pictures of some of the markings were taken using a HIROX KH-8700 3D Digital Microscope with an MXG-5000REZ triple objective revolving lens.

### Statistical analysis

Principal component analyses (PCA) were performed with R^125^. The cluster analysis was generated with PAST^126^ using a Manhattan pairwise similarity matrix. For these analyses, we used data from leporid assemblages in actualistic, experimental and archaeological studies. In the case of the actualistic and experimental studies, all data refer to leporid predators. The archaeological studies are of deposits and layers of the Upper Palaeolithic and Epipalaeolithic, where anthropic activity was especially intense. Anatomical data have been standardized using the minimal animal unit (MAU), expressed as a percentage^127^. Taphonomic and age of death data are expressed as percentages. References for all data used for comparative purposes can be consulted in Supplementary Tables S6–S8.

### Coprolite analysis

A total of 148 coprolite remains was studied from layer IIIa. The Cova del Gegant coprolites were analysed adapting the standardized method proposed by Jouy-Avantin *et al.*^128^ and Sanz *et al*.^129^. The analysis focused on the following aspects: preservation (complete, fragmentary and shapeless), morphotype (morphotype 1 to 3), morphometric on single droplets (recording maximum length and width or diameter in well preserved specimens) and bone content. The statistics derived from these data were compared with measurements reported in several studies on both fossil and modern scats^88,129,130^. For bone content analysis, the outer (external) surface or the sections of fragmented coprolites were examined to identify bone or tooth inclusions and where possible they were assigned to taxonomic and anatomical groups. Coprolites were not disaggregated in order to identify these items. Due to the high degree of fragmentation we used the taxonomic categories: large mammals (larger than leporids), mesofauna (leporids) and small mammals (smaller than leporids). Undetermined remains were recorded as skeletal tissues, such as shaft (long bone) or epiphysis (spongy) fragment^129^.

## Supplementary information

Supplementary Materials.

Supplementary Tables.

## Data Availability

All necessary permits were obtained from the *Departament de Cultura of the Generalitat de Catalunya* and from the local authorities for the excavation work at the Cova del Gegant under the direction of M.S. and J.D. for the study described here, which complied with all relevant regulations.
