# Supplementary material for: Taphonomic criteria for identifying Iberian lynx dens in quaternary deposits
Source: Sci Rep. 2020 Apr 29;10:7225. doi: 10.1038/s41598-020-63908-6 (PMC7190858; doi:10.1038/s41598-020-63908-6)
Supplement: Supplementary file 1 — Supplementary Materials. [file 41598_2020_63908_MOESM1_ESM.docx]

Taphonomic criteria for identifying Iberian lynx dens in quaternary deposits

**Antonio Rodríguez-Hidalgo ^1, 2, 3,^ *, Montserrat Sanz ^4^, Joan Daura ^4^, Antonio Sánchez-Marco ^5^**

^1^ Departamento de Prehistoria, Historia Antigua y Arqueología, Facultad de Geografía e Historia, Universidad Complutense de Madrid, Madrid, 28040, Spain

^2^ Instituto de Evolución en África (IDEA), Madrid, 28010, Spain

^3^ Institut Catalá de Paleoecología Humana i Evolució Social (IPHES). Tarragona, 4300, Spain

^4^ Grup de Recerca del Quaternari (GRQ)-SERP, Departament d’Història i Arqueologia, Universitat de Barcelona, Barcelona, 08001, Spain

^5^ Institut Català de Paleontologia Miquel Crusafont, Campus de la UAB, Bellaterra, 08193, Spain


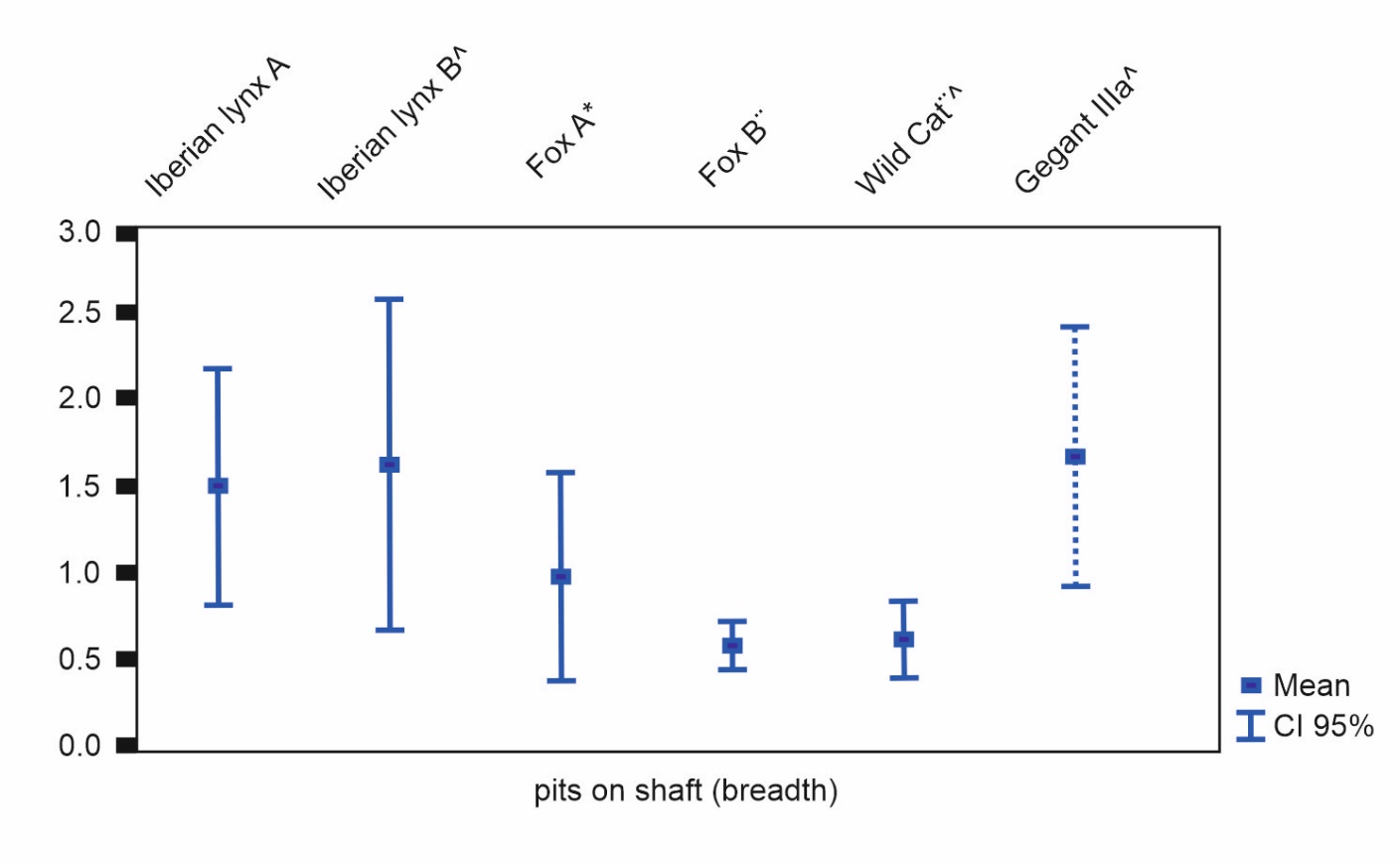


***Supplementary Figure S1. Comparisons of pits and punctures in bones of different lynx species, small felids, small canids and in Cova del Gegant IIIa remains. Mean and confidence interval (95%) of the dimensions of pits and punctures in cortical bone in millimetres. Revised data from Iberian lynx A***[*^20^*](https://paperpile.com/c/lRUA0f/MWwlM)***, Iberian lynx B***[*^29^*](https://paperpile.com/c/lRUA0f/nDEbf)***, Fox A***[*^117^*](https://paperpile.com/c/lRUA0f/HQ0nu)***, Fox B***[*^52^*](https://paperpile.com/c/lRUA0f/ty0h)***, Wild Cat***[*^52^*](https://paperpile.com/c/lRUA0f/ty0h)***. *Tooth pits on ungulate bones, ¨pits in cortical and thin cortical differentiated, ^less than 30 marks***.


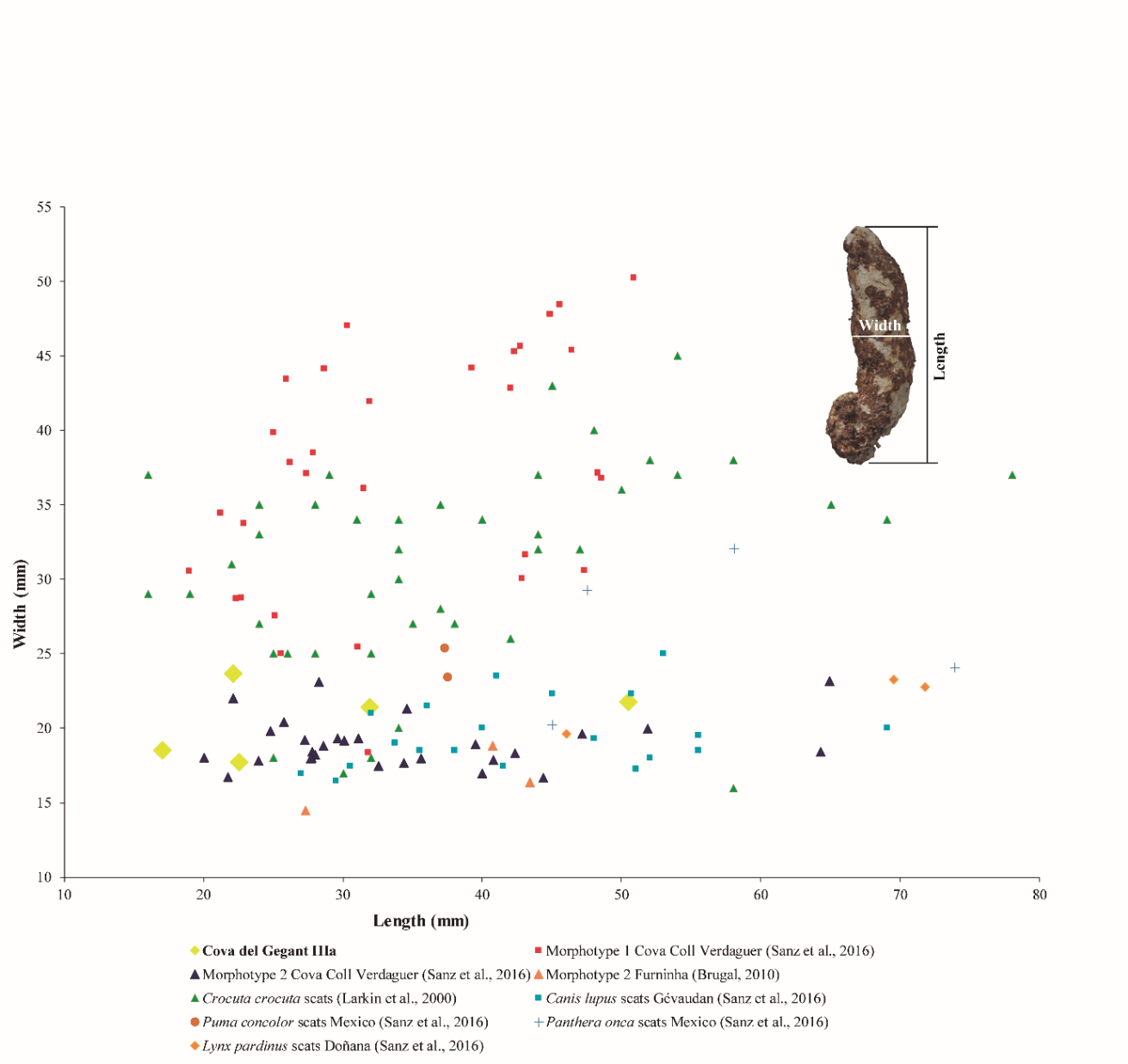


**Supplementary Figure 2. Cova del Gegant coprolites (morphotype 2) from layer IIIa, morphotypes from other Pleistocene sites and modern faeces morphometric comparative analysis.**

| **Indices** | **Percent (%)** | **Interpretation** |
| --- | --- | --- |
| PCRT/CR | 341.78 | Much More Post-Cranial |
| PCRAP/CR | 296.22 | Much More Post-Cranial |
| PCRLB/CR | 282.38 | Much More Post-Cranial than limbs |
| HU+FM/CR+MD | 155.44 | More Upper Limbs than Skull |
| TA/MD | 159.22 | More Tibia (NISP) than Mandible |
| TAE/MDE | 127.59 | More Tibia (Elements) than Mandible (MNE) |
| AUT/ZE | 250.46 | Much More Autopodium |
| Z/E | 131.91 | More Zygopodium |
| AN/PO | 54.22 | Balanced |
| HU/FM | 72.79 | More Humerus |
| RDU/TA | 50.61 | Balanced |
| MCP/MTT | 32.27 | More Metatarsal |
| W/L | 51 | Balanced |
| P/D | 57.4 | Slightly more distal |
| CO/LB | 22.7 | More limbs |

**Supplementary Table S1. Proportions of different parts of the leporid and bird’s skeleton in the assemblage.** **PCRT/CR**, total numbers of postcranial (limb elements, vertebrae and ribs) compared to total numbers of cranial elements (mandibles, maxillae and teeth);**PCRAP/CR**, appendicular skeleton elements (long bones, scapula, innominate, patella, metapodials, carpals, tarsals and phalanges) compared to cranial elements (mandibles, maxillae and teeth); **PCRLB/CR**, postcranial long bones (humerus, radius, ulna, femur and tibia) compared to cranial elements (mandibles and maxillae); **HU+FM/CR+MD,** stylopodium (femur and humerus) compared to cranial elements (mandible and maxillae); TA/MD, tibia NISP compared to mandible NISP; **TAE/MDE**, tibia MNE compared to mandibles NME; **AUT/ZE**, autopodium elements (metapodials, carpals, tarsals and phalanges) compared to zeugopodium and stylopodium (tibia, radius, ulna, humerus, femur and patellae); **Z/E**, zeugopodium elements (tibia, radius and ulna) compared to stylopodium (femur and humerus); **AN/PO**, humerus, radius, ulna and metacarpals compared to femur, tibia and metatarsals; **HU/FM**, humerus compared to femur; **RDU/TA**, radius, ulna compared to tibia; **MCP/CTT**, metacarpal compared to metatarsal; **W/L**, wing (humerus, radio/ulna, carpometacarpus) compared to legs (femur, tibiotarsus, tarsometatarsus); P/D proximal (coracoid, scapula, humerus, femur, tibiotarsus) compared to distal (radio/ulna, carpometacarpus, tarsometatarsus), CO/LB , core (coracoid, sternum, scapula) compared to limbs (humerus, radio/ulna, carpometacarpus, femur, tibiotarsus, tarsometatarsus)

|  | **C** |  | **PE** |  | **PES** |  | **S** |  | **SDE** |  | **DE** |  | **Total** |  |  |  |  |
| --- | --- | --- | --- | --- | --- | --- | --- | --- | --- | --- | --- | --- | --- | --- | --- | --- | --- |
|  | **N** | **%** | **N** | **%** | **N** | **%** | **N** | **%** | **N** | **%** | **N** | **%** |  |  |  |  |  |
| **Humerus** | 13 | 13.13 | 17 | 17.17 | 8 | 8.08 | 7 | 7.07 | 40 | 40.4 | 14 | 14.14 | 99 |  |  |  |  |
| **Radius** | 4 | 4.82 | 15 | 18.07 | 28 | 33.73 | 8 | 9.64 | 28 | 33.73 | 0 | - | 83 |  |  |  |  |
| **Ulna** | 0 | - | 13 | 20.63 | 16 | 25.4 | 19 | 30.16 | 6 | 9.52 | 9 | 14.29 | 63 |  |  |  |  |
| **Femur** | 17 | 12.5 | 29 | 21.32 | 41 | 30.15 | 15 | 11.03 | 23 | 16.91 | 11 | 8.09 | 136 |  |  |  |  |
| **Tibia** | 9 | 5.49 | 6 | 3.66 | 18 | 10.98 | 37 | 22.56 | 81 | 49.39 | 13 | 7.93 | 164 |  |  |  |  |
| **Metacarpal** | 86 | 71.07 | 3 | 2.48 | 22 | 18.18 | 0 | - | 6 | 4.96 | 4 | 3.31 | 121 |  |  |  |  |
| **Metatarsal** | 201 | 53.6 | 11 | 2.93 | 89 | 23.73 | 0 | - | 64 | 17.07 | 10 | 2.67 | 375 |  |  |  |  |
|  | 330 | 100 | 94 | 100 | 222 | 100 | 86 | 100 | 248 | 100 | 61 | 100 | 1041 |  |  |  |  |
| **Mandible** | **N** | **%** | **Cranium** | **N** | **%** | **Innominate** |  | **%** | **Scapula** | **N** | **%** | **Ribs** | **N** | **%** |  |  |  |
| **C** | 10 | 9.71 | C | 1 | 1.11 | C | 3 | 3.61 | C | 0 | - | C | 0 | - |  |  |  |
| **IP** | 6 | 5.83 | IB | 6 | 6.67 | A | 1 | 1.2 | GC | 6 | 18.75 | PE | 0 | - |  |  |  |
| **MBI** | 54 | 52.43 | IBM | 2 | 2.22 | AIS | 32 | 38.55 | GCN | 22 | 68.75 | PES | 50 | 46.73 |  |  |  |
| **MB** | 27 | 26.21 | M | 32 | 35.56 | AISIL | 12 | 14.46 | NF | 4 | 12.5 | S | 55 | 51.4 |  |  |  |
| **MBB** | 1 | 0.97 | ZA | 10 | 11.11 | AIL | 27 | 32.53 | F | 0 | - | SDE | 2 | 1.87 |  |  |  |
| **CP** | 5 | 4.85 | NC | 39 | 43.33 | IS | 4 | 4.82 |  | 32 | 100 |  | 107 | 100 |  |  |  |
|  | 103 | 100 |  | 90 | 100 | IL | 4 | 4.82 |  |  |  |  |  |  |  |  |  |
|  |  |  |  |  |  |  | 83 | 100 |  |  |  |  |  |  |  |  |  |
| **Patella** | **N** | **%** | **Car/Tar** | **N** | **%** | **Cal** |  | **%** | **Ast** | **N** | **%** | **Vertebrae** | **N** | **%** | **Phalanges** | **N** | **%** |
| **C** | 10 | 100 |  | 51 | 100 | C | 88 | 86.27 | C | 35 | 97.22 | C | 108 | 52.43 | C | 626 | 92.06 |
| **F** | 0 | 0 |  | 0 | 0 | F | 14 | 13.73 | F | 1 | 2.78 | F | 98 | 47.57 | P | 8 | 1.18 |
|  | 10 | 100 |  | 51 | 100 |  | 102 | 100 |  | 36 | 100 |  | 206 | 100 | D | 46 | 6.76 |
| **Teeth** | **“In situ”** | | | | |  | **Isolated** | | | | | |  |  |  | 680 | 100 |
|  | **Incisors** |  | **Upper Molars** |  | **Lower Molars** |  | **Incisors** |  | **Upper Molars** |  | **Lower Molars** |  |  |  |  |  |  |
|  | **N** | **%** | **N** | **%** | **N** | **%** | **N** | **%** | **N** | **%** | **N** | **%** |  |  |  |  |  |
| **C** | 19 | 82.61 | 57 | 100 | 250 | 100 | 106 | 96.36 | 124 | 82.67 | 194 | 82.91 |  |  |  |  |  |
| **F** | 4 | 17.39 | 0 | 0 | 0 | 0 | 4 | 3.64 | 26 | 17.33 | 40 | 17.09 |  |  |  |  |  |

**Supplementary Table S2. NISP and percentages of parts of the skeleton of leporids in each breakage category at Layer IIIa of Cova del Gegant.** Long bones, metacarpal and metatarsal bones were classified as complete (C), proximal epiphysis (PE), proximal epiphysis + shaft (PES), shaft (S), shaft + distal epiphysis (SDE) and distal epiphysis (DE). Mandible as complete (C), incisive part (IP), mandible body + incisive part (MBI), mandible body (MB), mandible body + branch (MBB) and condylar process (CP). Cranium as complete (C), incisive bone (IB), incisive bone + maxilla (IBM), maxilla (M), zygomatic arch (ZA) and neurocranium (NC). Innominate as complete (C), acetabulum (A), acetabulum + ischium (AIS), acetabulum + ischium + illium (AISIL), acetabulum + illium (AIL), ischium (IS) and illium (IL). Scapula as complete (C), glenoid cavity (GC), glenoid cavity + neck (GCN), neck + fossa (NF) and fossa (F). Ribs as complete (C), proximal epiphysis (PE), proximal epiphysis + shaft (PES), shaft (S), shaft + distal epiphysis (SDE). Vertebrae as complete (C), vertebral body (VB), vertebral epiphysis (VE) and spinous process (SP). Phalanges as complete (C), proximal fragment (P), distal fragment (D) and fragment (F). Patella, carpal/tarsal, calcaneum, astragalus and teeth as complete (C) and fragment (F)

| **breakage categories** | | | | | | | | |
| --- | --- | --- | --- | --- | --- | --- | --- | --- |
| **Skull** | ***N*** | **%** | **Pelvis** | ***N*** | **%** | **Sternum** | ***N*** | **%** |
| **whole** | 0 | 0 | **Synsacrum + ilium–ischii–pubis** | 0 | 0 | **more 1/2 with rostrum** | 0 | 0 |
| **beak + brain case without back part** | 0 | 0 | **Ilium–ischii–pubis** | 0 | 0 | **less 1/2 with rostrum** | 0 | 0 |
| **brain case without back part** | 0 | 0 | **synsacrum** | 2 | 100 | **fragment without rostrum** | 1 | 100 |
| **brain case** | 0 | 0 | **acetabulum** | 0 | 0 |  |  |  |
| **beak** | 0 | 0 |  |  |  |  |  |  |
|  | **Whole** | | **Proximal part** | | **Distal part** | | **Shaft** | |
|  | ***N*** | **%** | ***N*** | **%** | ***N*** | **%** | ***N*** | **%** |
| **Scapula** | 0 | - | 8 | 100 | 0 | 0 | 0 | 0 |
| **Coracoid** | 7 | 32 | 7 | 31.8 | 6 | 27.3 | 2 | 9.1 |
| **Humerus** | 4 | 21.1 | 6 | 31.6 | 8 | 42.1 | 1 | 5.3 |
| **Radius** | 3 | 60 | 1 | 20 | 1 | 20 | 0 | - |
| **Ulna** | 4 | 10.8 | 20 | 54.1 | 11 | 29.7 | 2 | 5.4 |
| **Carpometacarpus** | 11 | 61.1 | 5 | 27.8 | 2 | 11.1 | 0 | - |
| **Femur** | 1 | 5.9 | 11 | 64.7 | 5 | 29.4 | 0 | - |
| **Tibiotarsus** | 0 | - | 4 | 14.8 | 22 | 81.5 | 1 | 3.7 |
| **Tarsometatarsus** | 7 | 32 | 6 | 27 | 9 | 41 | 0 | - |
|  | **Complete** | | | | **Fragment** | | | |
|  | ***N*** | | **%** | | ***N*** | | **%** | |
| **Phalanges** | 34 | | 100 | | 0 | | 0 | |
| **Vertebrae** | 17 | | 100 | | 0 | | 0 | |
| **Ribs** | 0 | | 0 | | 0 | | 0 | |

**Supplementary Table S3. NISP and percentages of parts of the skeleton included in each breakage category for bird remains at Layer IIIa of Cova del Gegant**

|  | **Macromammals** | **%** | **Leporidae** | **%** | **Ave** | **%** | **Data IIIa** | **%** |
| --- | --- | --- | --- | --- | --- | --- | --- | --- |
| **Burning** | 19 | 5.2 | 35 | 1.2 | 4 | 1.7 | 58 | 1.6 |
| **Chemical Corrosion** | 1 | 0.3 | 28 | 0.9 | 4 | 1.7 | 33 | 0.9 |
| **Concretion** | 93 | 25.3 | 251 | 8.3 | 2 | 0.9 | 346 | 9.4 |
| **Cut marks** | 0 | 0 | 1 | 0.03 | 0 | - | 1 | 0.03 |
| **Digestion** | 1 | 0.3 | 40 | 1.3 | 0 | - | 41 | 1.1 |
| **Dissolution** | 2 | 0.5 | 0 | - | 1 | 0.4 | 3 | 0.1 |
| **Manganese Black Stain** | 86 | 23.4 | 884 | 29.1 | 55 | 23.4 | 1025 | 27.9 |
| **Rodent Marks** | 0 | - | 7 | 0.2 | 0 | - | 7 | 0.2 |
| **Root Stain** | 41 | 11.1 | 330 | 10.9 | 10 | 4.3 | 381 | 10.4 |
| **Scratching** | 0 | 0 | 7 | 0.2 | 0 | - | 7 | 0.2 |
| **Sediment pressure** | 5 | 1.4 | 0 | - | 0 | - | 5 | 0.1 |
| **Tooth marks** | 2 | 0.5 | 40 | 1.3 | 1 | 0.4 | 43 | 1.2 |
| **Trampling** | 4 | 1.1 | 0 | - | 5 | 2.1 | 9 | 0.2 |
| **Weathering** | 58 | 15.8 | 266 | 8.8 | 20 | 8.5 | 344 | 9.4 |
| **Total NSP/NISP** | 368 | - | 3035 | - | 235 | - | 3673 | - |

**Supplementary Table S4. Number of Identified Specimens (NISP) or Number of Specimens (NSP) and percentage of the taphonomic modifications recorded at Cova del Gegant IIIa over macromammals remains (Macro), leporids, birds and whole assemblage (Data IIIa)**

| **Element** | **Modification** | **Location** |
| --- | --- | --- |
| Humerus | scoring | shaft |
| Humerus | scoring | shaft |
| Humerus | puncture / scoring | shaft/shaft |
| Humerus | puncture + pit opposite / digestion | near epiphysis |
| Humerus | pit | shaft |
| Humerus | pit | shaft |
| Radius | puncture in fracture edge | shaft |
| Radius | pit | shaft |
| Radius | pit | shaft |
| Radius | pit | shaft |
| Femur | pits / scores | Epiph. prox.+ shaft/shaft |
| Femur | scoring | metaph. prox. |
| Femur | puncture in fracture edge | shaft |
| Femur | pit | shaft |
| Femur | scoring | shaft |
| Tibia | puncture in fracture edge | shaft |
| Tibia | pit | shaft |
| Tibia | scoring | shaft |
| Tibia | puncture in fracture edge | shaft |
| Tibia | scoring | shaft |
| Scapulae | puncture | neck |
| Coxae | puncture | ilium exterior |
| Coxae | puncture + pit opposite | ischium |
| Coxae | puncture + pit opposite in acetabulum | pit in ischium |
| Coxae | puncture + pit opposite | acetabulum |
| Coxae | puncture | acetabulum |
| Coxae | puncture | acetabulum |
| Coxae | puncture ilium | pit ilium |
| Coxae | puncture in fracture edge | ilium neck |
| Coxae | puncture | ischium |
| Coxae | puncture | ischium |
| Coxae | pits opposite /scoring | ilium neck |
| Coxae | pits opposite | ilium neck |
| Metatarsus | pit | shaft |
| Phalange | pit | shaft |
| Calcaneus | pit | corpus |
| Calcaneus | scoring | shaft |

**Supplementary Table S5. Description of carnivore tooth marks on leporid bones at the Cova del Gegant IIIa assemblage**

| **Reference** | **Type** | **Sample** | **Key** | **Mandible** | **Cranium** | **Humerus** | **Radius** | **Femur** | **Tibia** | **Scapula** | **Innominate** | **Metacarpal** | **Metatarsal** | **Carpal** | **Vertebra** | **Rib** |
| --- | --- | --- | --- | --- | --- | --- | --- | --- | --- | --- | --- | --- | --- | --- | --- | --- |
| [29](https://paperpile.com/c/lRUA0f/nDEbf) | Experimental | Natal Den | Lynx | 41.51 | 45.28 | 28.3 | 64.15 | 43.4 | 100 | 9.43 | 67.92 | 75.47 | 93.87 | 88.68 | 58.87 | 11.48 |
| [20](https://paperpile.com/c/lRUA0f/MWwlM) | Experimental | Sample A | Lynx | 41.12 | 51.4 | 21.5 | 75.23 | 35.98 | 100 | 7.94 | 89.72 | 51.78 | 89.84 | 100 | 16.94 | 17.52 |
| [20](https://paperpile.com/c/lRUA0f/MWwlM) | Experimental | Sample B | Lynx | 5.21 | 10.42 | 0 | 26.04 | 0 | 88.54 | 0 | 0 | 21.88 | 100.26 | 83.33 | 21.35 | 0 |
| [131](https://paperpile.com/c/lRUA0f/Ebsu) | Archaeological | UP-E | Homo | 50.28 | 49.44 | 36.39 | 37.78 | 62.78 | 69.44 | 35.56 | 100 | 4.78 | 11.32 | 32.78 | 0.65 | 1.25 |
| [132](https://paperpile.com/c/lRUA0f/GPLT) | Archaeological | E | Homo | 71.9 | 80.58 | 60.54 | 47.73 | 66.32 | 53.31 | 69.21 | 100 | 13.76 | 22.06 | 47.52 | 4.85 | 5.32 |
| [100](https://paperpile.com/c/lRUA0f/CmCfy) | Archaeological | UP | Homo | 35.94 | 50.16 | 28.43 | 40.58 | 28.59 | 28.43 | 36.26 | 100 | 11.02 | 21.49 | 49.2 | 1.47 | 2.8 |
| [19](https://paperpile.com/c/lRUA0f/Uxi1C) | Actualistic | Nest-1 | Owl | 13.24 | 26.47 | 19.12 | 17.65 | 50 | 38.24 | 8.82 | 100 | 7.65 | 37.5 | 51.47 | 12.65 | 15.2 |
| [19](https://paperpile.com/c/lRUA0f/Uxi1C) | Actualistic | Nest-2 | Owl | 33.33 | 24.24 | 18.18 | 7.58 | 42.42 | 50 | 9.09 | 100 | 10.91 | 49.62 | 72.73 | 11.89 | 28.28 |
| [23](https://paperpile.com/c/lRUA0f/7Sj0o) | Actualistic | Nest-1P | Owl | 18.75 | 12.5 | 12.5 | 18.75 | 75 | 62.5 | 6.25 | 100 | 2.5 | 32.81 | 62.5 | 6.56 | 7.81 |
| [23](https://paperpile.com/c/lRUA0f/7Sj0o) | Actualistic | Nest-2P | Owl | 3.45 | 6.9 | 10.34 | 6.9 | 29.31 | 44.83 | 6.9 | 100 | 0.69 | 40.09 | 37.93 | 5.34 | 3.88 |
| - | Archaeological | - | Gegant | 38.5 | 22.1 | 31.4 | 25.2 | 33.6 | 49.1 | 12.4 | 46 | 9.8 | 31.9 | 45.1 | 2.4 | 1.9 |

***Supplementary Table 6. Anatomical data expressed in %MAU (percentage minimal animal unit) used in the principal component analysis (Fig. 5a). The anatomical representation of the different elements of the leporid skeleton recovered by various authors during experimental studies of different rabbit predators and archaeological assemblages of the Upper Palaeolithic (UP) and Epipalaeolithic(E)attributed to anthropic agent.***

| **Reference** | **Key** | **Assemblage** | **CM** | **Bu** | **Di** | **T/B** | **CLL** | **Tu** | **Ad** |
| --- | --- | --- | --- | --- | --- | --- | --- | --- | --- |
| [133](https://paperpile.com/c/lRUA0f/puG4) | Homo Arq | **Pont d'Ambon, 4** | 12.5 | 13.7 | 0.4 | 0.2 | 1.5 | 26.2 | 96 |
| [133](https://paperpile.com/c/lRUA0f/puG4) | Homo Arq | **Moulin du Roc, Bigarée** | 11.7 | 13.9 | 0 | 0 | 1.2 | 20.6 | 100 |
| [100,134](https://paperpile.com/c/lRUA0f/CmCfy+MSf3) | Homo Arq | **Picareiro, F+G/H** | 0 | 3.2 | 0 | 0 | 3.8 | 40.2 | 99.4 |
| [135](https://paperpile.com/c/lRUA0f/E909) | Homo Arq | **Moli del Salt, Sup** | 1.1 | 20.1 | 0.2 | 1.5 | 4 | 8.3 | 75 |
| [135](https://paperpile.com/c/lRUA0f/E909) | Homo Arq | **Moli del Salt, Asup** | 1.2 | 23.5 | 0.03 | 0.9 | 0 | 1 | 91 |
| [135](https://paperpile.com/c/lRUA0f/E909) | Homo Arq | **Moli del Salt, A** | 1.5 | 30.5 | 0.03 | 1.4 | 4 | 3.9 | 96.7 |
| [135](https://paperpile.com/c/lRUA0f/E909) | Homo Arq | **Moli del Salt, B1** | 2.6 | 28 | 0.4 | 1.9 | 0 | 7.4 | 87.5 |
| [135](https://paperpile.com/c/lRUA0f/E909) | Homo Arq | **Moli del Salt, B1.1** | 1.6 | 20.5 | 0 | 0 | 4 | 4.7 | 75 |
| [135](https://paperpile.com/c/lRUA0f/E909) | Homo Arq | **Moli del Salt, B2** | 2.8 | 9.7 | 0 | 2.3 | 0 | 12.5 | 75 |
| [136](https://paperpile.com/c/lRUA0f/DAB9) | Homo Arq | **Balma del Gai, III** | 1.2 | 23.4 | 0.07 | 0.9 | 0.5 | 30.6 | 92.3 |
| **this study** | Gegant | **Cova del Gegant, IIIa** | 0.03 | 1.2 | 1.3 | 1.3 | 7.8 | 0.03 | 63 |
| [15](https://paperpile.com/c/lRUA0f/BjPin) | Warren | **Warren** | 0 | 0 | 0 | 0 | 57.3 | 0 | 33.5 |
| [137](https://paperpile.com/c/lRUA0f/ApeC) | Fox | **Vulpes, Rochers** | 0 | 0 | 10 | 21.2 | 30.8 | 7.8 | 45.9 |
| [18](https://paperpile.com/c/lRUA0f/xojkg) | Lynx, Scat | **Lynx, Scat** | 0 | 0 | 97.2 | 0.3 | 2.6 | 0 | 21.4 |
| [19](https://paperpile.com/c/lRUA0f/Uxi1C) | Owl | **Bubo, nest 1** | 0 | 0 | 68.8 | 1.9 | 17.1 | 0 | 50 |
| [19](https://paperpile.com/c/lRUA0f/Uxi1C) | Owl | **Bubo, nest 2** | 0 | 0 | 65.6 | 1.3 | 10.8 | 0 | 50 |
| [23](https://paperpile.com/c/lRUA0f/7Sj0o) | Owl | **Bubo, nest 1P** | 0 | 0 | 76.4 | 1.7 | 7.1 | 0 | 4.8 |
| [21](https://paperpile.com/c/lRUA0f/hPIHY) | Eagle | **B. Eagle** | 0 | 0 | 31.2 | 2.3 | 51.7 | 0 | 41.4 |
| [16](https://paperpile.com/c/lRUA0f/15wjq) | Eagle | **G. Eagle** | 0 | 0 | 32 | 1.2 | 26.3 | 0 | 83.5 |

**Supplementary Table S7. Taphonomic and age of death data of the leporid remains used in the principal component analysis PCA (Fig. 5b). The taphonomic and age of death data come from different experimental studies of different rabbit predators and archaeological assemblages of the Upper Palaeolithic and Epipalaeolithic attributed to anthropic agent and are expressed in % of presence. Key classification in Figure 5b, % cut marks (CM); % burning (Bu); % digested (Di); % tooth/beak marks (T/B); % complete long limbs (CLL); % tubes (Tu); % of adult leporids (Ad).**

| **Reference** | **Assemblage** | **CM** | **Bu** | **Di** | **T/B** | **CLL** |
| --- | --- | --- | --- | --- | --- | --- |
| [133](https://paperpile.com/c/lRUA0f/puG4) | **Pont d'Ambon, 4** | 12.5 | 13.7 | 0.4 | 0.2 | 26.2 |
| [133](https://paperpile.com/c/lRUA0f/puG4) | **Moulin du Roc, Bigarée** | 11.7 | 13.9 | 0 | 0 | 20.6 |
| [100,134](https://paperpile.com/c/lRUA0f/CmCfy+MSf3) | **Picareiro, F+G/H** | 0 | 3.2 | 0 | 0 | 40.2 |
| [135](https://paperpile.com/c/lRUA0f/E909) | **Moli del Salt, Sup** | 1.1 | 20.1 | 0.2 | 1.5 | 8.3 |
| [135](https://paperpile.com/c/lRUA0f/E909) | **Moli del Salt, Asup** | 1.2 | 23.5 | 0.03 | 0.9 | 1 |
| [135](https://paperpile.com/c/lRUA0f/E909) | **Moli del Salt, A** | 1.5 | 30.5 | 0.03 | 1.4 | 3.9 |
| [135](https://paperpile.com/c/lRUA0f/E909) | **Moli del Salt, B1** | 2.6 | 28 | 0.4 | 1.9 | 7.4 |
| [135](https://paperpile.com/c/lRUA0f/E909) | **Moli del Salt, B1.1** | 1.6 | 20.5 | 0 | 0 | 4.7 |
| [135](https://paperpile.com/c/lRUA0f/E909) | **Moli del Salt, B2** | 2.8 | 9.7 | 0 | 2.3 | 12.5 |
| [136](https://paperpile.com/c/lRUA0f/DAB9) | **Balma del Gai, III** | 1.2 | 23.4 | 0.07 | 0.9 | 30.6 |
| this study | **Cova del Gegant, IIIa** | 0.03 | 1.2 | 1.3 | 1.3 | 0.03 |
| [15](https://paperpile.com/c/lRUA0f/BjPin) | **Warren** | 0 | 0 | 0 | 0 | 0 |
| [137](https://paperpile.com/c/lRUA0f/ApeC) | **Vulpes, Rochers (Den)** | 0 | 0 | 10 | 21.2 | 7.8 |
| [18](https://paperpile.com/c/lRUA0f/xojkg) | **Lynx, Scat** | 0 | 0 | 97.2 | 0.3 | 0 |
| [19](https://paperpile.com/c/lRUA0f/Uxi1C) | **Bubo, Nest 1** | 0 | 0 | 68.8 | 1.9 | 0 |
| [19](https://paperpile.com/c/lRUA0f/Uxi1C) | **Bubo, Nest 2** | 0 | 0 | 65.6 | 1.3 | 0 |
| [23](https://paperpile.com/c/lRUA0f/7Sj0o) | **Bubo, Nest 1P** | 0 | 0 | 76.4 | 1.7 | 0 |
| [21](https://paperpile.com/c/lRUA0f/hPIHY) | **B. Eagle; Nest** | 0 | 0 | 31.2 | 2.3 | 0 |
| [16](https://paperpile.com/c/lRUA0f/15wjq) | **G. Eagle; Nest** | 0 | 0 | 32 | 1.2 | 0 |
| [24](https://paperpile.com/c/lRUA0f/03EaC) | **Vulpes, Noningested 1a** | 0 | 0 | 0 | 9.5 | 0 |
| [24](https://paperpile.com/c/lRUA0f/03EaC) | **Vulpes, Scat 1b** | 0 | 0 | 100 | 1.7 | 0 |
| [24](https://paperpile.com/c/lRUA0f/03EaC) | **Vulpes, Scat 2** | 0 | 0 | 99.3 | 5.3 | 0 |
| [137](https://paperpile.com/c/lRUA0f/ApeC) | **Bubo, Pellet_Carry-le-Rouet** | 0 | 0 | 81.5 | 3.8 | 3.3 |
| [138](https://paperpile.com/c/lRUA0f/SYjp) | **Bubo, Nest Hautes-Alpes** | 0 | 0 | 3.7 | 10.4 | 2.8 |
| [20](https://paperpile.com/c/lRUA0f/MWwlM) | **Lynx, Noningested Aceb** | 0 | 0 | 0 | 1.5 | 0.5 |
| [29](https://paperpile.com/c/lRUA0f/nDEbf) | **Lynx, Noningested Frar** | 0 | 0 | 0 | 1.8 | 0 |
| no published | **Vulpes, Den PnyRo** | 0 | 0 | 15.7 | 2.7 | 0.4 |

**Supplementary Table S8. Taphonomic data of the leporid remains used in the cluster analysis (Figure 5c). The taphonomic data come from different experimental studies of different rabbit predators and archaeological assemblages of the Upper Palaeolithic and Epipalaeolithic attributed to anthropic agent and are expressed in % of presence. % cut marks (CM); % burning (Bu); % digested (Di); % tooth/beak marks (T/B); % complete long limbs (CLL); % tubes (Tu); % of adult leporids (Ad).**

| **Element** | **Large mammal** | **Leporid** | **Leporid/Small mammal** | **Small mammal** | **Undetermined** |
| --- | --- | --- | --- | --- | --- |
| **Tooth** |  | 10 |  | 2 |  |
| **Cranium** |  | 1 |  |  |  |
| **Mandible** |  | 2 |  |  |  |
| **vertebra** |  |  |  | 1 |  |
| **Coxae** |  | 1 |  |  |  |
| **Femur** |  | 1 |  |  |  |
| **Humerus** |  | 1 |  |  |  |
| **Radius** |  | 1 |  |  |  |
| **Ulna** |  | 4 |  |  |  |
| **Phalanx** |  | 1 |  |  |  |
| **Shaft fragment** | 5 | 23 | 9 | 4 | 3 |
| **Spongy bone** |  | 1 |  |  |  |
| **Charcoal** |  |  |  |  | 1 |
| **Total** | **5** | **46** | **9** | **7** | **4** |

**Supplementary Table 9. Bone contents from morphotype 2 coprolites**

# **Supplementary references**

131. [Martínez-Polanco, M. F., Blasco, R., Rosell, J., Ibañez, N. & Vaquero, M. Rabbits as food at the end of the Upper Palaeolithic at Molí del Salt (Catalonia, Spain). *Int. J. Osteoarchaeol.* **27**, 342–355 (2017).](http://paperpile.com/b/lRUA0f/Ebsu)

132. [Rosado-Méndez, N. Y., Lloveras, L., García-Argüelles, P. & Nadal, J. The role of small prey in hunter–gatherer subsistence strategies from the Late Pleistocene–Early Holocene transition site in NE Iberia: the leporid accumulation from the Epipalaeolithic level of Balma del Gai site. *Archaeol. Anthropol. Sci.* **11**, 2507–2525 (2019).](http://paperpile.com/b/lRUA0f/GPLT)

133. [Jones, E. L. Broad spectrum diets and the European rabbit (*Oryctolagus cuniculus*): dietary change during the Pleistocene-Holocene transition in the Dordogne, southwestern France. (University of Washington, 2004).](http://paperpile.com/b/lRUA0f/puG4)

134. [Bicho, N., Haws, J. & Hockett, B. Two sides of the same coin—rocks, bones and site function of Picareiro Cave, central Portugal. *J. Anthropol. Archaeol.* **25**, 485–499 (2006).](http://paperpile.com/b/lRUA0f/MSf3)

135. [Rufà, A., Blasco, R., Rosell, J. & Vaquero, M. What is going on at the Molí del Salt site? A zooarchaeological approach to the last hunter-gatherers from South Catalonia. *Hist. Biol.* **30**, 786–806 (2018).](http://paperpile.com/b/lRUA0f/E909)

136. [Rosado-Méndez, N. Y. The exploitation of small prey among the last hunter-gatherers in the Northeast of the Iberian Peninsula: the case of the leporids in the Epipalaeolithic. (Universitat de Barcelona, 2017).](http://paperpile.com/b/lRUA0f/DAB9)

137. [Cochard, D. Les léporidés dans la subsistance paléolithique du sud de la France. (Université Sciences et Technologies - Bordeaux I, 2004).](http://paperpile.com/b/lRUA0f/ApeC)

138. [Guennouni, K. E. Les lapins du Pléistocène moyen et supérieur de quelques sites préhistoriques de l’Europe méditerranéenne: Terra-Amata, Orgnac 3, Baume Bonne, grotte du Lazaret, grotte du Boquete de Zafarraya, Arma delle Manie : étude paléontologique, taphonomique et archéozoologique. (Paris, Muséum National d’Histoire Naturelle, 2001).](http://paperpile.com/b/lRUA0f/SYjp)
